# Macroecology and macroevolution of the latitudinal diversity gradient in ants

**DOI:** 10.1038/s41467-018-04218-4

**Published:** 2018-05-03

**Authors:** Evan P. Economo, Nitish Narula, Nicholas R. Friedman, Michael D. Weiser, Benoit Guénard

**Affiliations:** 1 Okinawa Institute of Science and Technology Graduate University, Onna, Okinawa 904-0495 Japan; 2 Department of Biology, University of Oklahoma, 730 Van Vleet Oval, Norman, OK 73019 USA; 3 School of Biological Sciences, University of Hong Kong, Pok Fu Lam, Hong Kong SAR China

## Abstract

The latitudinal diversity gradient—the tendency for more species to occur toward the equator—is the dominant pattern of life on Earth, yet the mechanisms responsible for it remain largely unexplained. Recently, the analysis of global data has led to advances in understanding, but these advances have been mostly limited to vertebrates and trees and have not provided consensus answers. Here we synthesize large-scale geographic, phylogenetic, and fossil data for an exemplar invertebrate group—ants—and investigate whether the latitudinal diversity gradient arose due to higher rates of net diversification in the tropics, or due to a longer time period to accumulate diversity due to Earth’s climatic history. We find that latitudinal affinity is highly conserved, temperate clades are young and clustered within tropical clades, and diversification rate shows no systematic variation with latitude. These results indicate that diversification time—and not rate—is the main driver of the diversity gradient in ants.

## Abstract (web summary)

Multiple hypotheses have been proposed for the declining biodiversity gradient between the tropics and poles. Here, the authors compile and analyze geographic data for all ant species and large-scale phylogenies, suggesting that diversification time drives the latitudinal diversity gradient in ants.

## Introduction

While many hypotheses have been proposed for the mechanisms underlying the latitudinal diversity gradient (LDG), distinguishing among them has proven to be a major challenge^1–4^. The recent emergence of comprehensive global datasets on the spatial distribution of species^5–7^ and the reconstruction of large-scale time-calibrated phylogenies has facilitated the global analyses of biodiversity gradients of vertebrates^8–10^ and woody plants^11^. However, these efforts have not produced consensus answers thus far, and the latitudinal diversification patterns of highly diverse invertebrate groups—notably insects—remain gaps in our knowledge. Pioneering studies of insect latitudinal diversification patterns have been conducted on specific insect groups (e.g., leaf beetles^12^ and swallowtail butterflies^13, 14^), but the data-deficiency of most insect taxa has precluded large-scale analyses on a scale comparable with vertebrate groups.

Here we present comprehensive geographic data encompassing nearly all described ant species**—**to our knowledge the first such dataset for any diverse terrestrial arthropod group**—**and reconstruct phylogenetic trees encompassing all the major ant clades. We integrate and analyze these datasets to test hypotheses explaining the striking latitudinal gradient in ant diversity. Ants are attractive as an exemplar insect group given their near-ubiquity in terrestrial ecosystems, their ecological dominance rivaling or exceeding most vertebrate groups, their relevance to economic and conservation concerns, and their high**—**but not intractably high**—**levels of species diversity^15^.

Numerous hypotheses have been proposed for the latitudinal diversity gradient^2–4^, but these can be broadly sorted into three “umbrella hypotheses”. First, the ecological regulation hypothesis (ERH) posits that there are equilibrial ecological limits to species number, which vary systematically with latitude, perhaps due to the direct influence of climate and/or available energy (e.g., refs. ^16–19^). Here, speciation and extinction rates may change over time to regulate diversity near those limits, but variation in those rates is not causally responsible for disparities in richness. Second, the diversification rate hypothesis (DRH) posits that speciation and/or extinction rates vary systematically with latitude due to some causal factor(s) and diversity gradients are a bottom-up consequence of this rate variation. Finally, the tropical conservatism hypothesis^1, 20, 21^ (TCH) is the idea that, in principle, extratropical ecosystems could support as high diversity as tropical regions and net diversification rates do not vary systematically with latitude, but colder climates are generally younger and have not had time to build-up diversity. We focus here on testing predictions of the latter two hypotheses (DRH and TCH), which are differentiated by whether the latitudinal gradient is due to disparities in the rate-of-diversification or time-for-diversification.

The TCH is based on the idea that before the Eocene, the Earth was thought to be much warmer than it is now^20^. Warm “megathermal” biomes, which today are limited to the tropics, are thought to have covered much of the Earth’s surface, including high-latitude areas, such as Europe^22^. At the onset of the Oligocene, climatic cooling opened up a vast expanse of cold-climate land area^23^. According to the TCH, evolutionary transitions between climatic zones are difficult and this limited the number of lineages successfully colonizing younger, colder areas. The low number of older lineages adapted to cold climates, combined with the reduced time for diversification^21^, explains the disparity in species diversity across latitudes observed today. The TCH has been recently tested in plants^11^, and birds^24^, with findings mostly consistent with the hypothesis. Ant climatic niches have been previously shown to be phylogenetically conserved^25^, and the ant fossil record supports the presence of present day “tropical” lineages at high latitudes in the Eocene^26^.

The DRH asserts that net diversification rate is higher in the tropics than the temperate zone, and the LDG is an epiphenomenon of differential accumulation rates. Many authors have proposed gradients in speciation rate, extinction rate, or both, that result in latitudinal diversification rate differences (reviewed in ref. ^3^). For example, one such explanation is that the higher temperatures in the tropics lead to higher mutation rates, increasing speciation rates^27^. Thus far, empirical tests to are mostly equivocal in their support for the DRH, with different studies giving conflicting results even within the same taxon (e.g., birds^9, 28–30^ and mammals^8, 28, 31^).

Both the DRH and TCH hypotheses depend on phylogenetic niche conservatism^32^ in adaptation to climate. This niche conservatism would be present if evolutionary transitions between climatic zones (tropical to temperate) are difficult because they require physiological adaptations to tolerate colder conditions, and such transitions are thus expected to be rare in the evolutionary history of a group. If climate tolerance evolves quickly, then the effects of differences in either historical age or net diversification rate on richness will be mediated by spillover from other latitudes.

If climatic niches are phylogenetically conserved enough to maintain richness gradients, the DRH and TCH are distinguished by whether those gradients are due to differences in time-for-diversification (TCH) or systematic variation in net diversification rate (DRH), either of which should leave a signature on a phylogeny. The TCH makes the additional prediction that most temperate diversification should have occurred in the last 34 my since the Oligocene cooling^20^.

To test these predictions, we examined the macroecology and macroevolution of global ant diversity using newly assembled comprehensive geographic and phylogenetic datasets. We compiled and curated a database of the geographic distributions of all 14,912 described ant species and subspecies, using a synthesis of published literature, museum databases, and online repositories. We then reconstructed a set of all-ant phylogenies to complement these geographic data. Recent studies have established the main features of the backbone of the ant tree of life, as well as structure within the 16 extant ant subfamilies^33–37^. We extended these efforts by reconstructing backbone trees combining the most recently available molecular data (as of mid-2015), and using those trees as a basis to construct dated phylogeny sets that place all ant species into a phylogenetic context while reflecting phylogenetic uncertainty given the fact that most species lack molecular data. As the timescale of ant evolution has been controversial in the past, we made a vigorous effort to date the tree using recently developed methods that incorporate a wide range of fossil evidence. In particular, we performed a large-scale implementation of the fossilized birth-death process^38^, dating the tree with over 500 fossil taxa from a comprehensive database of ant fossils, in addition to more traditional node-calibration approaches. As it is probable that the tropical ant species are undersampled relative to extratropical species, we also evaluate the sensitivity of each of our results to this potential bias.

## Results

### Species richness and phylogenetic diversity

**Fig. 1:**
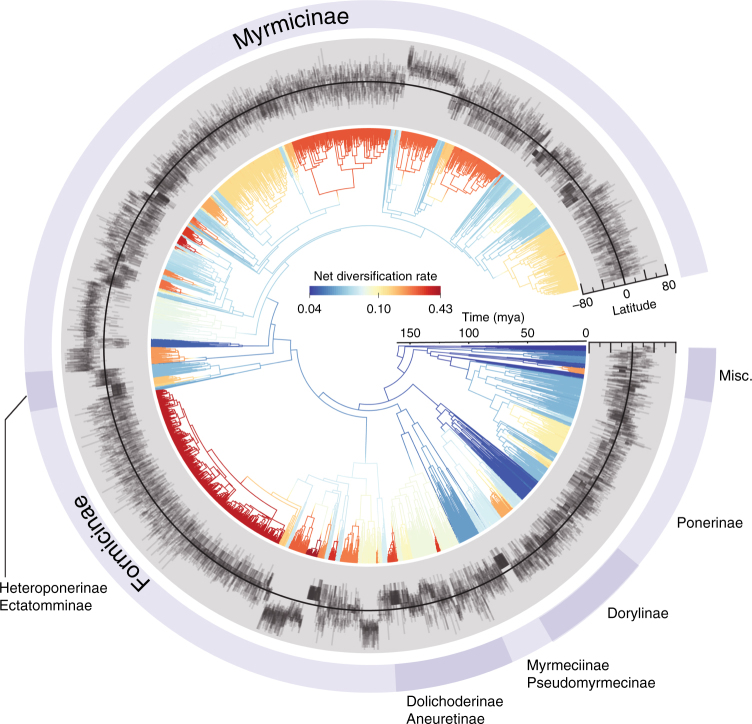
Phylogenetic position and latitudinal extent of 14,512 ant species. The all-ant ML phylogeny dated using median node ages across the posterior tree set from a Bayesian dating analysis, annotated with the latitudinal ranges of each species. Branches are colored by net diversification rate inferred by BAMM^62^. The displayed tree was constructed using median node ages under the NC-stem dating/grafting method and was used to give typical results for visualization purposes, but the analyses were performed individually on 100 separate trees from the posterior for each method

The phylogenetic analyses recovered the main known relationships^33–36^ among ant subfamilies and among clades within subfamilies (Fig. 1, Supplementary Fig. 1). While results based on other methodological variations are presented in the supplementary figures, we present representative results (using the NC-stem dating/grafting method, see Supplementary Note 1) in the main text.

**Fig. 2:**
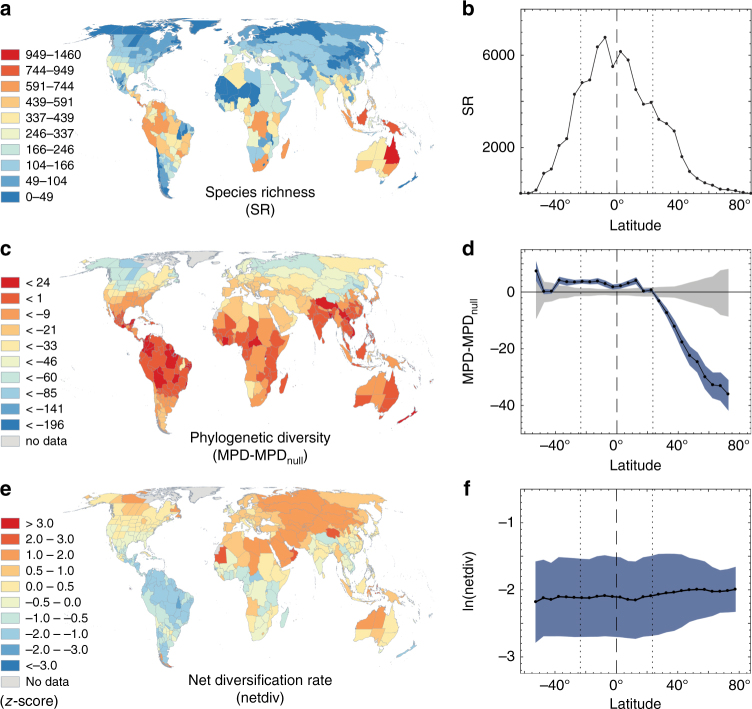
Global patterns of ant diversity and evolution. Species richness by **a** region and **b** 5° latitudinal band. Phylogenetic diversity by **c** region and **d** latitudinal band, where the gray area reflects the distribution of 95% null values while the blue area reflects 95% of observed values across 100 trees in the posterior distribution. Both null and observed values were subtracted from the null mean for each tree. Mean of ln(median diversification rate across trees) for all species in each **e** region or **f** latitudinal band, with blue shading standard deviation of the variation within each band. These plots are based on trees constructed with NC-stem dating/grafting method, but corresponding results with other methods are found in Supplementary Figs. 2-3

As expected, ant species richness peaks in tropical latitudes (Fig. 2), consistent with patterns observed in many other taxa as well as previous work on ants^39, 40^. Phylogenetic diversity strongly decreases with increasing latitude in the northern hemisphere, indicating that northern temperate lineages are clustered into relatively few clades (Fig. 2). We confirmed this pattern is robust to dating and grafting methods, and uncertainty in the phylogeny (Supplementary Fig. 2). The strong north-south hemispheric asymmetry to this pattern was present in both the east and west hemispheres (Supplementary Fig. 2).

### Diversification rate

We inferred diversification rates to be highly variable across ant lineages (Fig. 1); however, we found no evidence of a systematic negative relationship between net diversification rate and latitude (Fig. 2, Supplementary Figs. 3-4), as predicted by the DRH. This result was consistent across dating methods, whether we used clade grafting or incomplete sampling fractions to represent undiscovered ant diversity (see Supplementary Note 2), and whether we used clade-wise phylogenetic regressions (i.e., PLGS) or lineage-wise structured rate permutations (i.e., STRAPP) to test for correlations (Supplementary Fig. 4). Most of the analyses actually showed a weak marginally significant/insignificant positive latitude-diversification rate trend (i.e., higher diversification rates outside the tropics). We performed two tests to assess whether undersampling of tropical species could obscure a latitude-diversification rate correlation (see Supplementary Note 3), and both found this to be unlikely given reasonable assumptions about undiscovered ant species in the tropics. We found that this bias could, however, be responsible for the weak positive correlation observed in our analyses (Supplementary Fig. 5).

### The timing of tropical and extratropical diversification

**Fig. 3:**
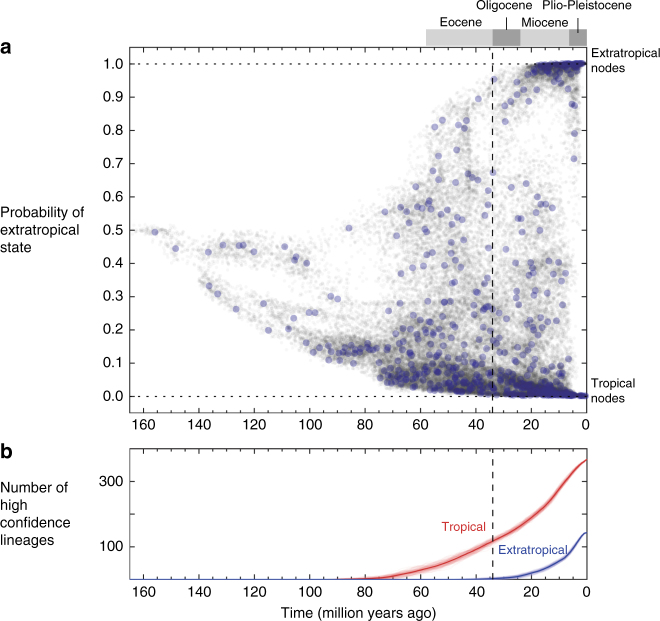
Tropical and extratropical ant lineages through time. **a** The marginal probability each node was extratropical in the backbone trees (NC dating method) under the AIC minimizing trait evolution model. Gray dots are nodes from all 100 posterior trees, and blue dots are nodes from the mcc tree. **b** The number of lineages through time reconstructed with high confidence (probability >0.90) to be either tropical (red) or extratropical (blue) for each posterior tree (lighter color) and the mean across trees (darker color). Results for other trait-evolution models are presented in Supplementary Figs. 6,7

Our data support the TCH prediction that, in addition to being phylogenetically clustered, extratropical diversification was concentrated after the Eocene-Oligocene transition 34 million years ago. Ancestral state estimations on the backbone trees show that there are few or no lineages reconstructed with high confidence to be extratropical that are older than 34 million years, while such “high-confidence” tropical lineages existed as early as 80 mya (Fig. 3, Supplementary Figs. 6-7). Analyses of time-dependent latitudinal trait evolution models are consistent with a recent acceleration of evolution into the extratropics, with the optimal timing of this increase during the Miocene (means: 19.5 mya, 16.9 mya for NC and FBD methods, respectively, Supplementary Fig. 7). However, while all the models reconstruct a relative youth of extratropical lineages compared to tropical lineages, it is difficult to statistically distinguish whether this pattern is due to asymmetric evolution (with more frequent transitions from the extratropics into the tropics) or more symmetric, but accelerating evolution (Supplementary Figs. 6-7). Further discussion of the different models and results is presented in Supplementary Note 4.

## Discussion

Our analysis found that ant lineages outside the tropics are younger and more phylogenetically clustered, but are diversifying at similar (or rather, similarly heterogeneous) rates compared with those inside the tropics. This combination of results is more consistent with the TCH than the DRH in explaining the latitudinal gradient in ant diversity.

The strong phylogenetic clustering of high-latitude species and clades is an indication of niche conservatism; it is difficult to transition to colder high-latitude climates, and thus it happens relatively rarely. We note again, however, that this pattern is highly asymmetric: northern latitude ant faunas are highly clustered, while southern high-latitude ant lineages tend to be derived from the same clades as tropical species. This hemispheric asymmetry is strikingly similar to the pattern recently reported in New World trees^11^ and echoes hemispheric asymmetries in species richness for ant assemblages^40^ among other taxa^41^. This may reflect the fact that cold, high-latitude climates in the northern hemisphere are harsher and more seasonal compared with cold climates in the southern hemisphere^41^.

Our data also match predictions of the TCH related to the timing of transitions into high-latitude regions. Notably, there are few lineages that are reconstructed to have an extratropical affinity older than the Oligocene transition (34 mya). However, epoch models found the optimal breakpoint to be in the mid-Miocene. Although, the Oligocene transition was our a priori prediction based on previous work, we also note that the mid-Miocene was also a period of rapid cooling^42^. Future studies on ants and other taxa should evaluate whether the Miocene was actually a more pertinent time period for increased evolutionary transitions from the tropics to the temperate zone.

The lack of a correlation between diversification rate and latitude argues against the simple explanation that the gradient is a result of differential rates of accumulation. Speciation and/or extinction could vary with latitude, but our results suggest such a relationship is not the primary driver of the gradient through an effect on differential accumulation rates. That said, our analyses focus on deeper timescales and evolutionary differences among ant clades, and lack phylogenetic resolution within the 262 terminal clades, some of which are large and themselves exhibit a latitudinal gradient. It is possible that diversification rate varies with latitude within these clades, and analyses at the level of all ants are not at the relevant taxonomic or phylogenetic scale. In a parallel study^43^, we addressed this by analyzing the evolution of the LDG using a well-resolved global phylogeny of the hyperdiverse genus *Pheidole* (the second largest ant genus with >1000 described species), which diversified and evolved a latitudinal gradient within the past 30 my. We again found no systematic correlation between diversification rate and latitude, and showed that niche conservatism and tropical ancestry are sufficient to explain the gradient in *Pheidole*. Moreover, while it is not possible to test for ecological regulation in our ant-wide phylogenies given how they were constructed, there was evidence of diversity regulation in *Pheidole*.

Our study gives a macroscopic view of ant diversity by synthesizing much of the taxonomic, phylogenic, fossil, and geographic data generated since Linnaeus described the first ant species in 1758^44^. However, this view is still blurry: our knowledge of ant biodiversity, like all other insect groups, is far from complete. Inventory efforts need to be pursued for at least another generation until large-scale biodiversity patterns, and thus our understanding of their origins, can be considered settled. Nonetheless, the current evidence suggests that the overall gradient arises due to niche conservatism interacting with Earth’s geologic history on long timescales, but also may involve ecological regulation on shorter timescales. There is no hint of a negative diversification rate—latitude correlation in ants across any of our analyses, a finding which challenges a major class of hypotheses that link the gradient to systematic differences in macroevolutionary rates. This finding also accords with recent high-resolution analyses on swallowtail butterflies^14^, which found a strong historical effect on the diversity gradient. Further work examining smaller clades in great detail, combined with large macro-taxonomic studies like the one presented here, will provide complementary insights into the origins of the gradient in insects and other highly diverse invertebrate taxa.

The latitudinal biodiversity gradient has been a particular challenge for science to explain, mostly because of number of confounding factors that could plausibly affect diversity patterns on the one planet known to harbor life. However, the emergence and synthesis of new geographic and phylogenetic datasets across an increasingly broad range of organisms, combined with quantitative methods that probe deeply into their patterns, provides a pathway to a deeper understanding of the most general geographic pattern of life on Earth.

## Methods

### Species distributional data

Our geographic data were retrieved from a newly assembled comprehensive dataset on ant species distributions, the Global Ant Biodiversity Informatics (GABI) database^45^. The dataset is a compilation of ant species geographic records from all available sources, combining museum records, specimen databases, and data from over 8500 publications. The construction of the database and all the supporting issues are described in a dedicated publication^45^; however, we briefly summarize the process here.

The main sources of data include published literature, online databases, and museum collections, totaling approximately 1.7 million species occurrence records. While the database is continuously updated to reflect new publications, we used the version available in August 2015 for this study, with legacy taxonomy was updated using AntCat.org^46^ at that point. Studies describing taxonomic changes in ants are published on a nearly weekly basis and a broad analysis like this one must necessarily lag behind the latest taxonomic and phylogenetic developments. Our taxonomy generally follows mid-2015 and thus does not reflect taxonomic changes since then. However, we made one exception by splitting *Colobopsis* from *Camponotus*, making both monophyletic, following a recent revision of the subfamily Formicinae^47^.We made an exception as this affects the largest ant genus in one of the most diverse clades, and is thus potentially important for our analysis. We treated subspecies as full species for the purposes of analysis, and refer to them in our study here as “species”.

The dataset includes many historical records with varying degrees of geographic precision. For many species, few or no point records are available, as geo-coordinates have been a fairly recent addition to collecting practices. To facilitate analysis and allow for the inclusion of the most records and species, we assigned each occurrence record for the 14,912 species and subspecies to a system of 415 polygons covering Earth’s surface. The polygons reflect a mixture of political (e.g., country, first administrative level) and geologic units, such as islands, and were chosen opportunistically to match how authors and collectors recorded data. Although, the geometry of these areas is somewhat arbitrary, it should not affect the broad global patterns at the scale we investigate. Records were curated for quality and plausibility, and records were classified as native or exotic. Only native ranges and records that were not flagged as dubious in the database were included in the analysis. In total, this gave 70,816 species by polygon incidences. The GABI dataset can be visualized through the web-mapping tool antmaps.org^48^. Map data in this paper were plotted with QGIS (www.qgis.org).

### Phylogenetic tree-set reconstruction

We sought to reconstruct phylogenetic tree sets that represent current knowledge of ant phylogenetic history and integrate over uncertainty due to the fact that most species lack molecular data. Because, the details are voluminous, we provide a summary here and the full phylogenetic methods in Supplementary Note 1. We consolidated sequence data from recent studies^33–35, 37, 49–53^ (Supplementary Data 1), aligned a set of 11 nuclear loci using MACSE^54^ and MAFFT^55^, and set nucleotide substitution and clock models using Partition Finder^56^ and ClockstaR^57^. We reconstructed a set of dated backbone trees using a combination of RaxML^58^ and BEAST2^59^ and a database of >500 fossil taxa. We used both conventional Node Calibration (NC), testing different choices of prior distribution (Supplementary Fig. 8), and Fossilized Birth-Death^38^ (FBD) approaches, with the latter implemented using a set of monophyly constraints (Supplementary Data 2) and the sampled ancestor plugin^60^ for BEAST2. To reconstruct trees representing all ant taxa (with uncertainty) we performed a clade grafting procedure. Using 100 backbone trees from the posterior for each dating method and known taxonomic and phylogenetic relationships, we grafted 262 terminal clades (Supplementary Data 3, Supplementary Fig. 9) onto the backbone trees to produce posterior tree sets with 14,512 taxa included.

### Phylogenetic diversity

We estimated the mean pairwise distance (MPD) statistic to test for phylogenetic diversity patterns with latitude, which we used as an indicator of niche conservatism of latitudinal range. MPD is appropriate given the nature of our all-ant phylogenies, in which each posterior tree has randomly resolved topology within the 262 terminal clades. As MPD measures clustering of a set of species across the entire phylogeny, it is not very sensitive to the details of recent divergences across species. The main reason is that 97% of species pairs in our dataset include species from two different terminal clades, and in such pairs within-clade topological uncertainty is irrelevant. Rather, the pattern will be driven by geographic differences in how many clades are present in a particular area (i.e., if only a few clades reach high latitudes, more pairs will be within-clade vs. among clade relative to the whole tree) and the phylogenetic relationships among clades (i.e., whether high-latitude clades are themselves clustered on the tree). Further discussion of how phylogenetic uncertainty and sampling bias may affect our analysis of phylogenetic diversity is presented in Supplementary Note 3.

We calculated phylogenetic diversity (MPD) both for species grouped into latitudinal bands as well as for species occurring in each polygonal area using the *picante* package^61^ in R. For each species the minimum and maximum latitude of their distribution was estimated from the geographic dataset. For a given species, this was the minimum and maximum latitude overall geographic polygons in which the species is known to occur. For calculating the phylogenetic diversity of latitudinal bands, we calculated MPD on the species whose latitudinal range intersected with the band, while MPD for each polygon was found using the pool of species that occur there. Null distributions for each assemblage (either latitudinal band or polygonal area) and tree from the posterior set were found by sampling a random set of species matching the richness of each observed assemblage 100 times and calculating MPD. The latitudinal band analyses were repeated for the entire globe and for the New World and Old World individually.

### Diversification rate inference and statistical tests

We sought to infer lineage-specific rates of diversification from our phylogenies, and test for correlations with latitude. Because the details of these analyses are voluminous, we provide a summary here and full description and discussion in Supplementary Note 2. We used BAMM^62^ to perform Bayesian inference of net diversification rates across our set of 200 all-ant phylogenies (100 with each grafting method), using constant-rate macroevolutionary models and focusing on net diversification (not speciation and extinction). We also ran BAMM on the backbone trees directly using clade-specific sampling fractions rather than the all-ant trees. We tested for latitudinal-dependency of diversification rate on the posterior (from BAMM) of each tree in our tree sets using both clade-wise phylogeny-corrected regressions (i.e., PGLS^63^) and lineage-wise structured rate permutation tests (i.e., STRAPP^64, 65^). Because the performance of BAMM is under debate^66, 67^, we also performed tests to validate our results with other methods (see Supplementary Note 2). For the clade-wise PGLS, we additionally devised a weighting scheme to preferentially weight clades with less uncertainty in diversification rate (due, in part, to the fact that clades ages had different degrees of uncertainty). We further tested for the effect of potential latitudinal sampling bias (i.e., more undiscovered tropical taxa relative to extratropical) using two tests (1) either thinning different fractions of tropical species from the analysis or (2) adjusting incomplete sampling fractions and rerunning BAMM and the statistical analyses (see Supplementary Note 3).

### Ancestral state estimation

We estimated ancestral states with parametric models of discrete character evolution. In other studies, latitudinal affinity has been treated alternatively as continuous or a discrete character. We chose to treat tropical affinity as a discrete character for the ancestral state estimation for the following reasons. First, the ranges of the majority of species were either all within the tropics or all outside of the tropics. If we treat the fraction of the range in the tropics as an unbounded continuous measure, this means the values are bounded and most are located at extremes, which makes confidence intervals difficult to interpret. If we use midpoint latitude as a continuous variable, as was used in some previous studies, we were concerned ancestral states would be expected to tend to the tropics by chance alone, because ancestral state reconstructions tend toward (weighted) average values in deeper time (even if associated with increasing uncertainty) and the tropics are intermediate of all latitudinal ranges. Thus, we decided treating tropical affinity as a binary trait (midpoint latitude either inside or outside the tropics) was a more conservative approach and best able to represent uncertainty in our reconstructions. We interpret “tropical” and “extratropical” ancestral states to reflect affinity for climates we now associate with those regions, rather than literal location of ancestors. For these analyses, we used the backbone tree set, not full all-ant trees, because reconstructing ancestral states depends on the precise branching structure and using randomized terminal clades would lead to inference of artificially high rates of character evolution. We also pruned morphospecies from the backbone phylogenies, because our geographic dataset is based on valid species, which left 512 taxa on the trees.

We estimated ancestral states using continuous-time Markov models of discrete trait evolution (i.e., Mk2 models). These are governed by transition rates from the tropics to the extratropics (*q*_tr→ex_), and from the extratropics to the tropics (*q*_ex→tr_). We considered both symmetric (*q*_tr→ex_ = *q*_ex→tr_) and asymmetric (*q*_tr→ex_ ≠ *q*_ex→tr_) models. We attempted to fit both time-homogeneous and “epoch” models of trait evolution^68^, where transition rates shift at some time point—the boundary between two epochs. In practice, the asymmetric epoch model was problematic to fit, so we excluded it (see Supplementary Note 4).

We used the R package diversitree^69^ to fit the models of trait evolution with maximum likelihood and reconstruct marginal ancestral state probabilities under the ML model. The two-epoch model was fit by sweeping across possible epoch boundary times at 1 my intervals between 1 and 120 mya, fitting an 2-epoch model at each potential breakpoint. For each tree, we calculated a likelihood profile across possible epoch boundary locations, with the profile being the increase in likelihood of the 2-epoch model over the time-homogeneous model. We compared models with the Akaike Information Criterion (AIC), which takes into account that the symmetric time-homogeneous, asymmetric time-homogeneous, and 2-epoch symmetric models have 1, 2, and 3 parameters, respectively.

For the time-homogeneous models, we crosschecked the marginal ancestral state probabilities from diversitree with marginal probabilities calculated from stochastic character mapping simulations using the make.simmap function in the R package *phytools*, and found them to be identical aside from sampling noise. We also performed a resampling procedure to evaluate whether the ancestral state pattern we recovered was sensitive to small sample size (Supplementary Note 3, Supplementary Fig. 10)

To find the number of lineages with a given state at each time point (used in the LTT plots), we interpolated these probabilities along each branch to calculate the probability the lineage was tropical or extratropical at each time point. The interpolation was based on a function developed for mapping traits on trees^70^, which takes into account the estimated marginal ancestral state probabilities at nodes at each end of a branch in question, and the distances between time point being considered on the branch and the bounding nodes. The time points used were 1 million year intervals starting at the present up to the root age estimated in the backbone trees rounded up.

### Data availability

A list of extant and fossil taxa used in phylogenetic analyses and GenBank accession codes are provided in Supplementary Data 1. The DNA sequence alignment, control files for the phylogenetic and BAMM analyses, posterior samples of the dated backbone trees and grafted all-ant phylogenies across method permutations, and species by polygon incidence matrix are archived in a Dryad repository (doi:10.5061/dryad.g579t7k). Current versions of the GABI dataset can be visualized through the web portal antmaps.org.

## Electronic supplementary material

Supplementary Information

Description of Additional Supplementary Files

Supplementary Data 1

Supplementary Data 2

Supplementary Data 3
