# Supplementary material for: Macroecology and macroevolution of the latitudinal diversity gradient in ants
Source: Nat Commun. 2018 May 3;9:1778. doi: 10.1038/s41467-018-04218-4 (PMC5934361; doi:10.1038/s41467-018-04218-4)
Supplement: Supplementary file 1 — Supplementary Information [file 41467_2018_4218_MOESM1_ESM.pdf]

Supplementary Information

Macroecology and macroevolution of the latitudinal diversity gradient in ants

Economo *et al.*

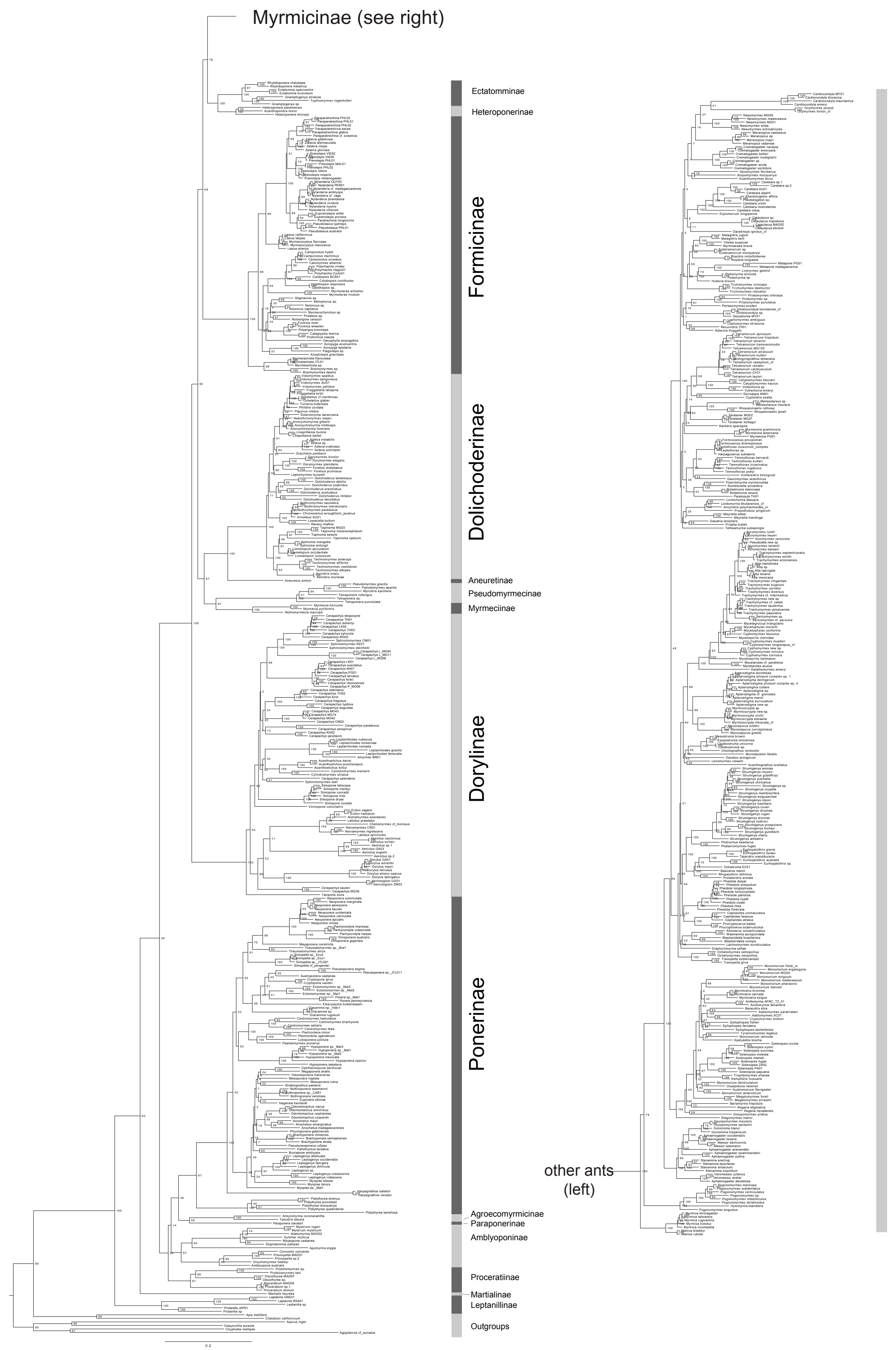

Supplementary Figure 1 | Maximum Likelihood backbone phylogeny inferred with RaxML based on 11 loci. This topology was used for the node calibration (NC) dating analysis, and highly supported nodes were used to justify monophyly constraints for the FBD dating analysis in which topology was not otherwise fixed. Node numbers reflect bootstrap support. Scale in substitution per unit branch length.

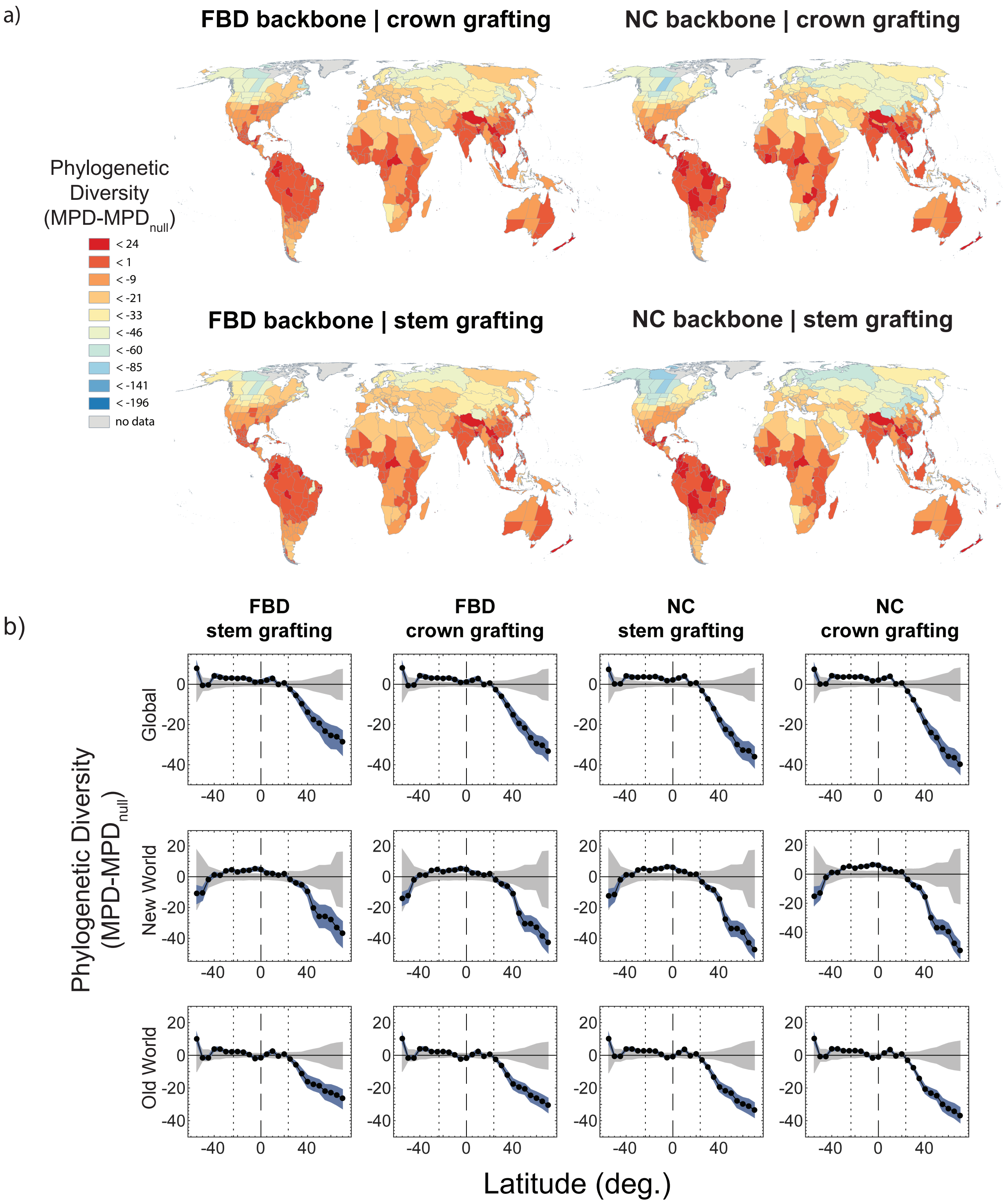

Supplementary Figure 2 | Global patterns in ant phylogenetic diversity. Mean phylognetic diversity (MPD) for a) each polygonal area and b) latitudinal band across 100 trees in the posterior tree set for the two backbone dating methods (FBD and NC) and grafting methods (stem and crown). For both, the observed MPD was subtracted from the null mean (which varies slightly among trees). The gray shaded area represents the null distribution and blue reflects the 95% of the observed values across trees.

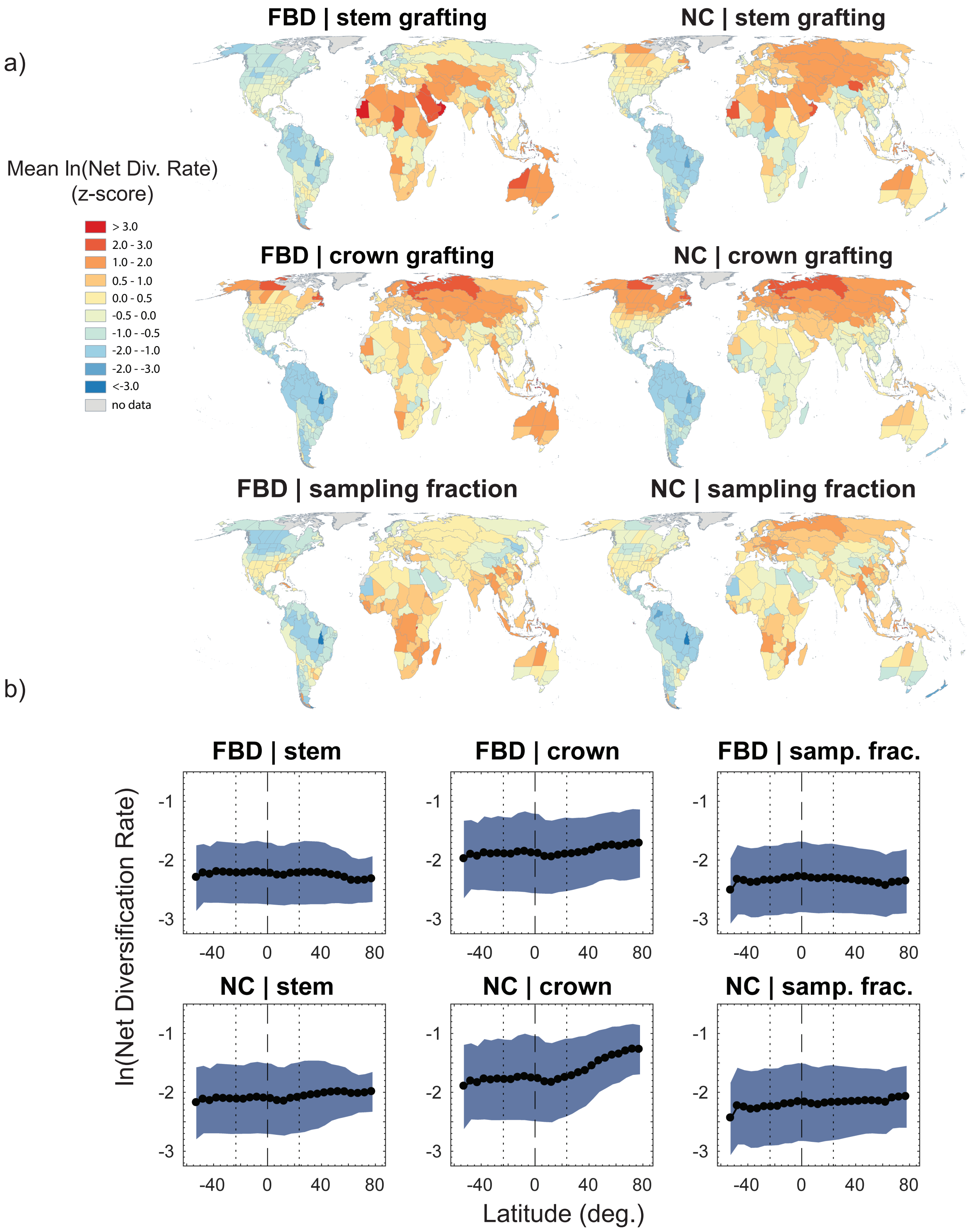

Supplementary Figure 3 | Net diversification of ant lineages across latitudes. Mean of species' median ln(net diversification rates) taken over all species occurring within a) each polygonal area and b) each latitudinal band for each method combination. For a given species, the species median was taken across over the estimate inferred from each of 100 trees in the posterior sample. In b), the black line is the median for the band and the blue shaded area represents the standard deviation across all species. FBD and NC refer to the two dating methods, stem and crown to the two grafting methods. We also inferred net diversification with BAMM directly on the backbone using the clade-specific sampling fraction option.

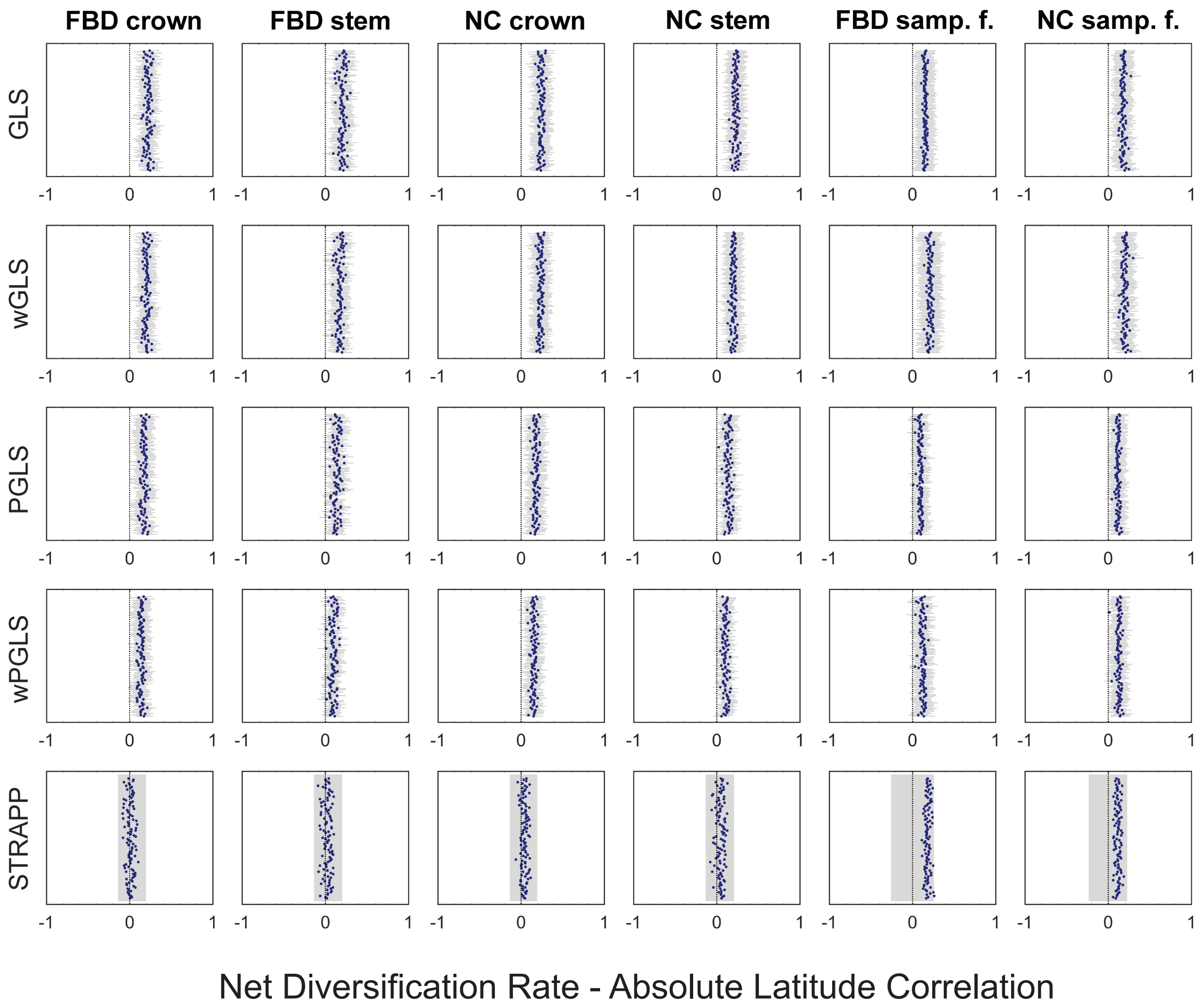

Supplementary Figure 4 | Standardized correlation coefficients between ln(net diversification rate) and absolute latitude across methodologies. GLS, wGLS, PGLS, wPGLS, refer to clade-wise regressions (n=262) with different weighting schemes (see Supplementary Note 2), where each blue dot is the standardized correlation coefficient on one tree from the posterior, and bar is the 95% CI. The results are stacked vertically to display variation among posterior trees. STRAPP refers to the lineage-based permutation test, and here the blue dot is the Pearson correlation coefficient, and the shaded area encloses 95% null distributions from 1000 replicates. The columns represent combinations of different tree dating methods (FBD or NC) and different methods of inferring net diversification rate (stem or crown grafting, or using BAMM directly on the backbone phylogeny with clade-specific sampling fractions).

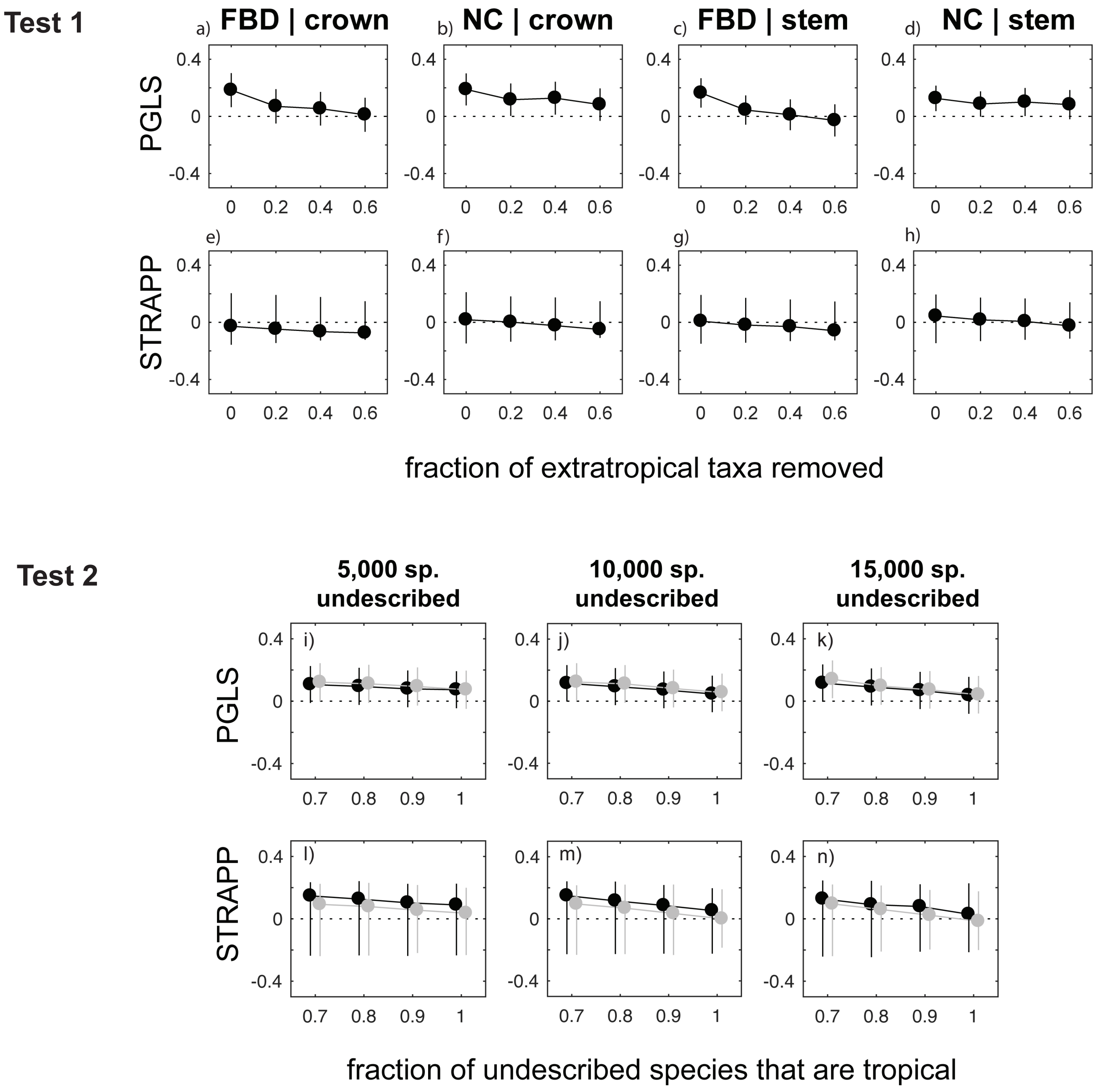

Supplementary Figure 5 | Sensitivity test of the diversification rate-latitude relationship to potential latitudinal sampling bias. a-h) In test 1, we pruned different fractions of extra-tropical species off of the grafted all-ant trees to compensate for potential oversampling in the extratropics, and inferred diversification rates for different dating/grafting method combinations. In test 2, we used the backbone trees and adjusted the clade-specific sampling fraction to reflect both uncertainty in the number of undescribed ant species (5K-15K) and the fraction of those that are tropical (0.7-1.0). In top rows (a-e, i-k) , the dot is the standardized coefficient of the clade-wise PGLS with bars reflecting 95%CI, and in the bottom rows (e-h, l-n) the dots reflect the Pearson correlation and bars the 95% null distribution from the structured rate permutation test. For i-n) the black and gray dots represent correlations and null distributions for diversification used for the NC and FBD backbone trees, respectively (the x values are slightly offset for visibility).

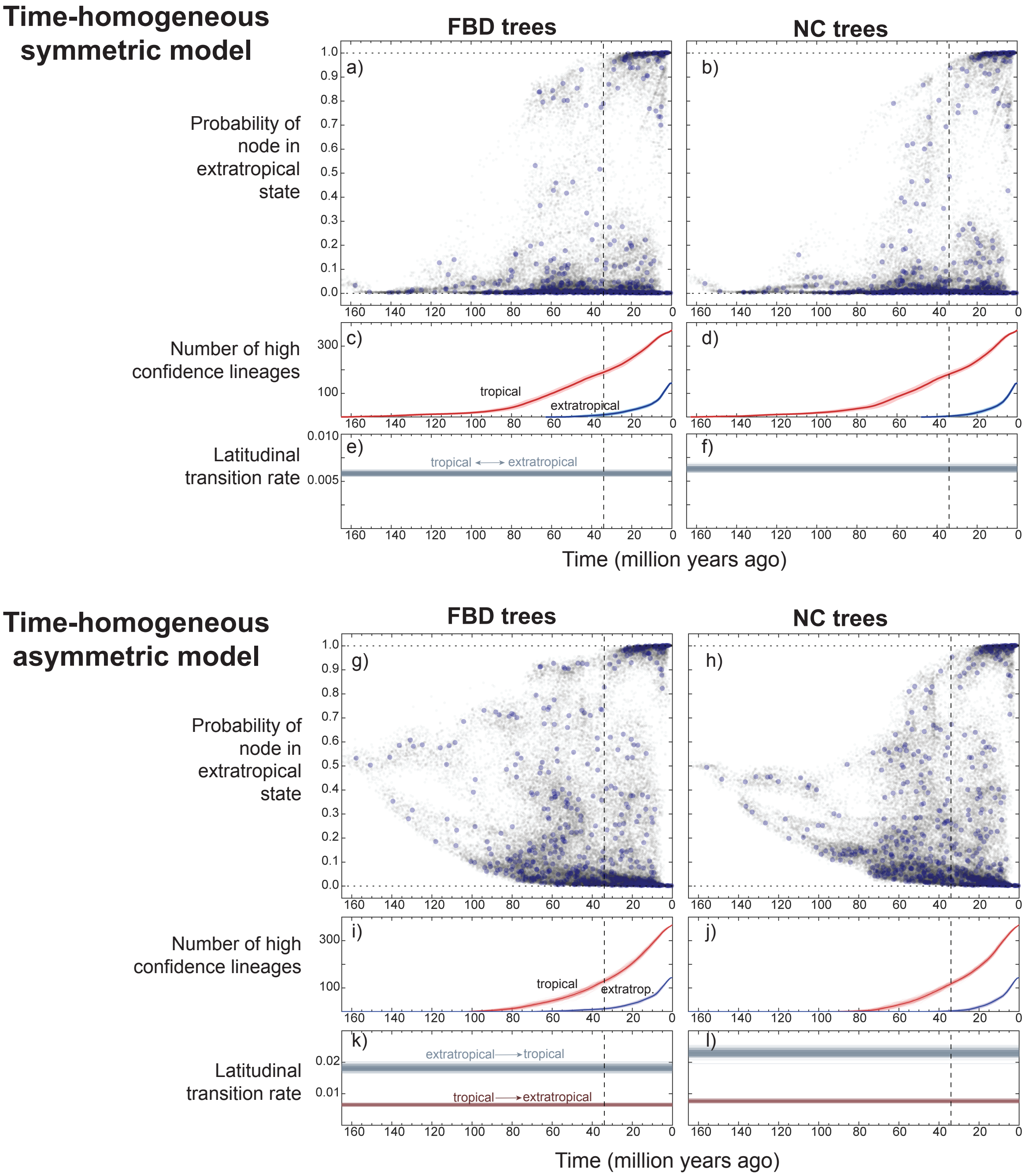

Supplementary Figure 6 | Ancestral state estimation and lineages through time under time-homogeneous models of trait evolution. a,b,g,h) Ages and estimated ancestral state probabilities for nodes in the backbone trees. Gray points are nodes from individual trees in the posterior, blue points are from the mcc tree of each method. c,d,i,j) The number of lineages inferred with over 90% chance of being either tropical or extratropical at each time point. e,f,k,l) The maximum likelihood rate(s) of latitudinal trait evolution. Both of these are time-homogeneous rates of trait evolution, but a-d) depicts symmetric models where transitions happen at equal rates in both directions, and e-l) asymmetric models which fit different rates to transitions in each direction. The asymmetric model was strongly favored over the symmetric model for all trees in both posterior tree sets (FBD: ΔAIC ~12.0, NC: ΔAIC~16.7).

Epoch model

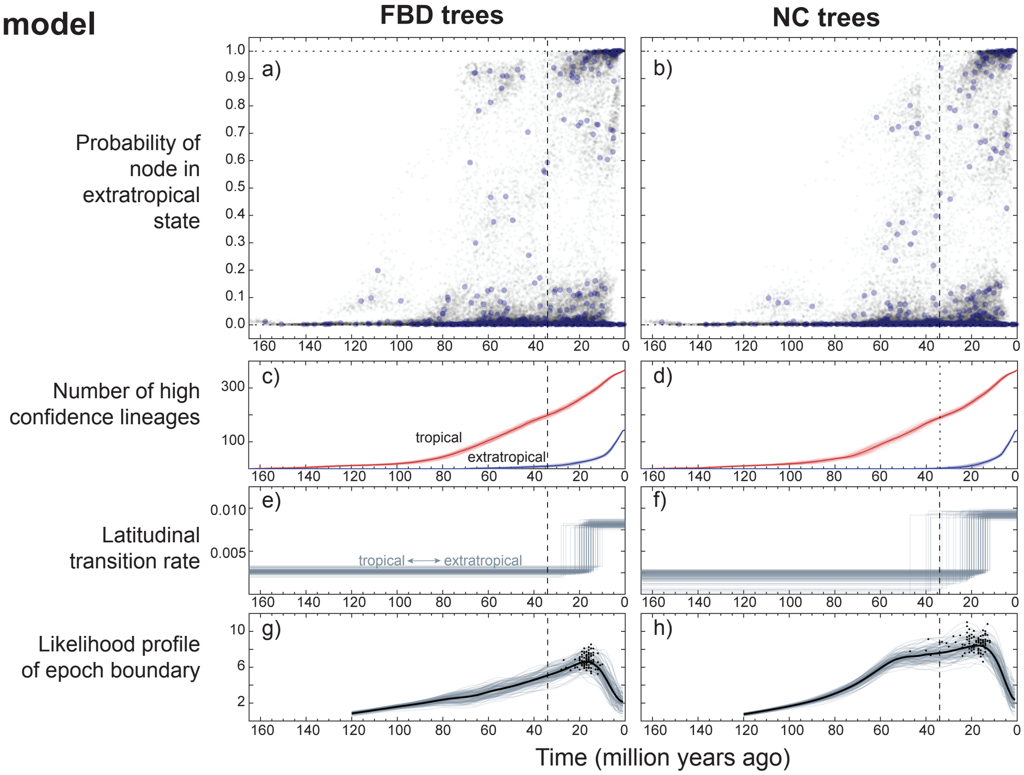

Supplementary Figure 7 | Ancestral state estimation and lineages through time under a symmetric two-epoch model of trait evolution. a,b) Node ages and estimated ancestral state probabilities. Gray dots are nodes from individual trees in the posterior, blue dots are from the mcc tree of each method. c,d) The number of lineages inferred with over 90% chance of being either tropical or extratropical over time. e,f) The maximum likelihood rates of latitudinal trait evolution. g,h) The likelihood profile for each posterior tree (increase in log-likelihood of 2-epoch symmetric model over time-homogeneous symmetric model for a given tree) for models fitted by sweeping across different epoch boundary times, with black dots the ML times for each tree and black line the average profile across posterior trees. The symmetric epoch model outperformed the symmetric time homogeneous model and was slightly worse fit than the time-homogenous asymmetric model (FBD: ΔAIC~2.5, NC: ΔAIC=3.3). All models agree on there being few high-confidence extratropical lineages reconstructed to be older than the Oligocene, with most arising since the Miocene.

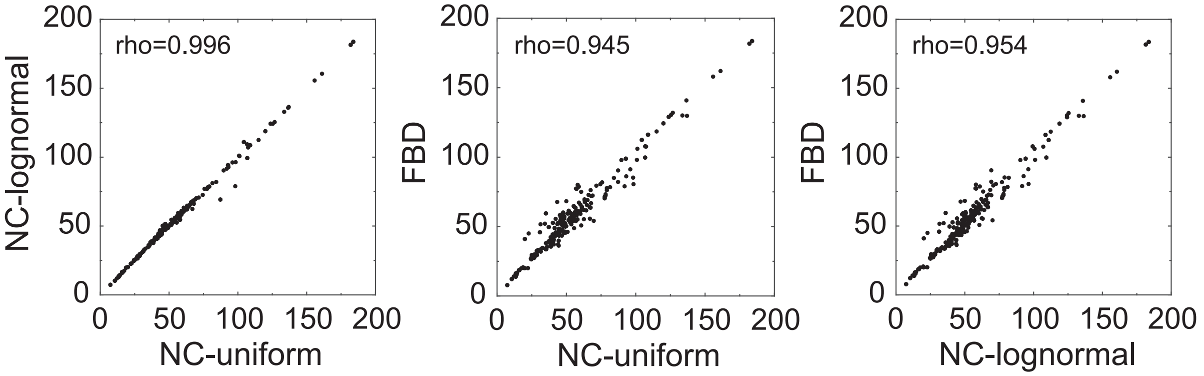

Supplementary Figure 8 | Comparison of node ages from different tree dating methods. Pairwise omparison and Spearman's rho of median node ages estimated with the three dating methods across 800 posterior trees each. NC-uniform refers to nodes calibrated with uniform prior distributions, NC-lognormal se lognormal distributions as node calibrations, and FBD is the fossilized birth-death process. The node ages were calculated for 277 nodes that appear in all trees.

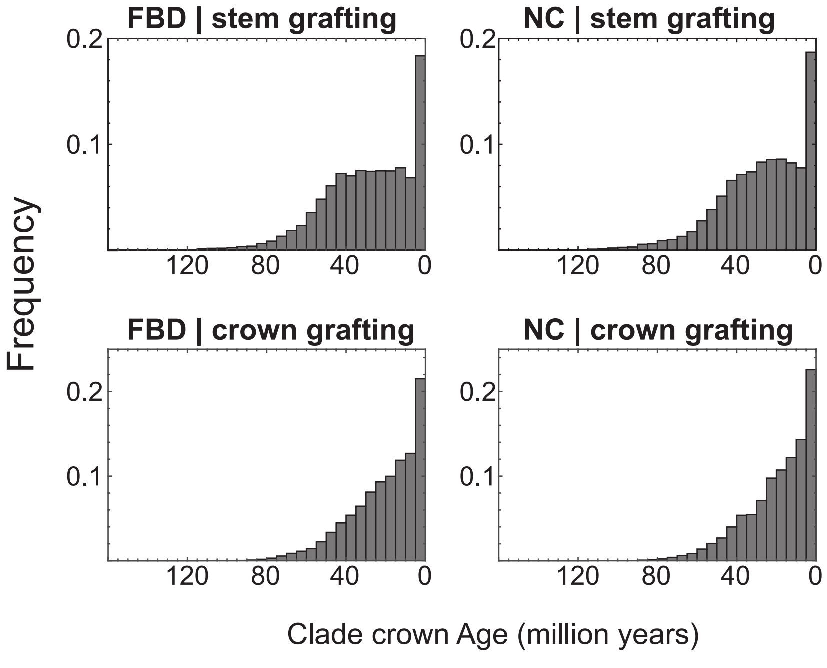

Supplementary Figure 9 | The distribution of terminal clade ages used in the grafting step. The distribution of terminal clade crown ages (after grafting) across 100 trees from each combination of the the two dating and grafting methods. Terminal clades with only one taxon (thus no crown age) were coded as zeros. This indicates the timescale of the grafting process.

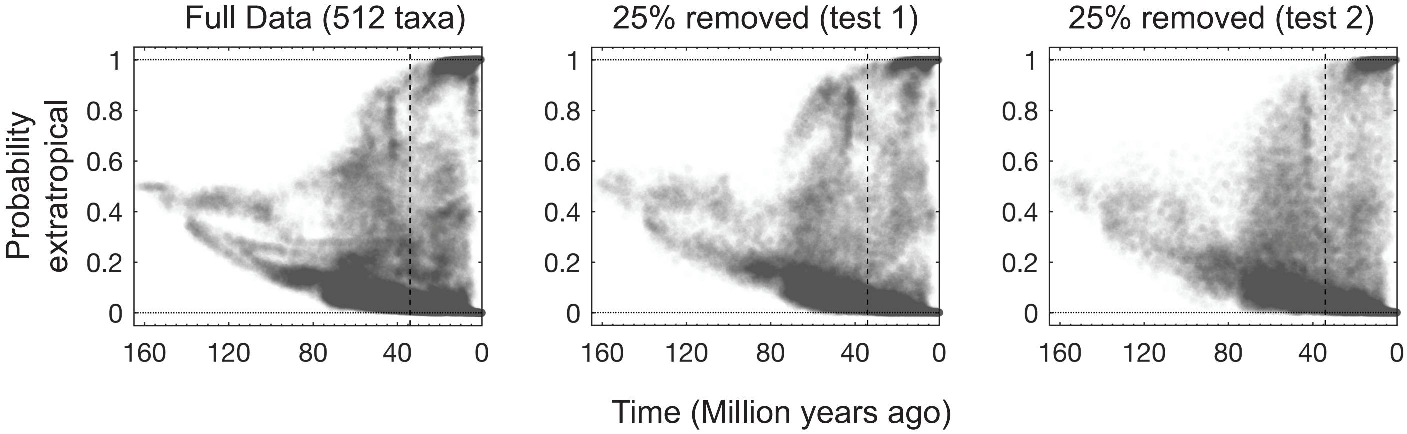

Supplementary Figure 10 | Sensitivity test of ancestral state estimation to sample size. To test if the ancestral state estimation was sensitive to sample size, we dropped 25% of taxa and recalculated the ancestral state pattern in Figure 3. a) Ancestral state pattern recovered across 100 posterior trees using the full data (NC dating). b-c) Ancestral states reconstructed on a reduced dataset after dropping 25% of taxa from all trees. The two tests differ in that in test 1, the same 25% of taxa were dropped from all trees, while in test 2, a different random set was dropped from each tree.

Supplementary Note 1: Phylogenetic Reconstruction Methods

Overview. We sought to reconstruct phylogenetic tree sets that represent current knowledge of ant phylogenetic history while integrating over uncertainty due to the fact that most species lack molecular data. In this regard, we follow similar efforts reconstructing comprehensive phylogenies for plants,[1] birds,[2] and mammals[3] representing taxa both with and without molecular data.

Recent studies have established the main backbone of the ant tree of life,[4–8] with molecular data available for all ant subfamilies and most genera. Subfamily and genus-level taxonomy has been extensively reorganized to be consistent with phylogenetic relationships, and with a few notable exceptions (e.g. *Monomorium*, *Cerapachys*), are mostly monophyletic (or a combination of a few genera are monophyletic) and taxonomy can be used to place them on the tree. Given this, our approach was to a) first reconstruct backbone phylogenies with a broad set of data representing the main ant lineages, b) date the backbone trees using a dataset of 509 fossil taxa, and then c) identify well-supported clades that can be grafted onto the appropriate backbone lineages leaving trees with all (or nearly all) ant taxa represented. We discuss each of these steps in turn. The alignment, ML tree, BEAST xml files, dated backbone posterior tree sets (with and without fossil taxa), and all-ant posterior trees generated with the different method permutations are all available in a Dryad repository (doi:10.5061/dryad.g579t7k).

Molecular Data and Taxon Selection. We retreived nucleotide sequences provided by eight different ant phylogenetic studies as either nexus alignments from Treebase[9] or as FASTA sequences from GenBank.[10] The eight studies include Brady et al. (2006),[5] Moreau et al. (2006),[4] Schultz and Brady (2008),[11] Ward et al. (2010),[6] LaPolla et al. (2010),[12] Schmidt (2013),[13] Brady et al. (2014),[14] and Ward et al. (2015).[8] We chose to use alignments from published studies rather than mine genetic data from GenBank directly, because we have found some GenBank data to represent misidentified loci and/or taxa, and believe those used in published phylogenetic studies are most reliable. Data in nexus alignment files were de-aligned to retrieve the nucleotide sequences and converted into FASTA format. The full dataset included 7,283 sequences representing 13 loci, and 948 taxa representing 687 ant species from 276 genera and 16 Formicidae subfamilies. It also included 43 sequences from six outgroup taxa obtained from the Schmidt 2013 study.[13] With the help of AntCat.org,[15] we manually updated taxonomic names to represent the latest

taxonomic changes and to correct misidentifications. Finally, we filtered the dataset to maximize coverage and eliminate redundancy which reduced the dataset to 5,465 sequences representing 11 loci, 673 ant species (269 genera) and six outgroup taxa. The 11 genetic loci are identified as follows: *18S ribosomal RNA* (*18S*), *28S ribosomal RNA* (*28S*), *abdominal-A* (*abdA*), *arginine kinase* (*argK*), *carbamoyl-phosphate synthetase 2* (*CAD*), *elongation factor 1-alpha F1* (*EF1aF1*), *elongation factor 1-alpha F2* (*EF1aF2*), *long-wavelength rhodopsin* (*LwRh*), *DNA topoisomerase* (*Top*), *ultrabithorax* (*Ubx*), *wingless* (*wg*). GenBank accession numbers for all sequences as well as detailed taxonomic information are available in Supplementary Data 1.

Nucleotide Alignment. Since nine out of the 11 loci are protein-coding genes, we generated codon-aware alignments for these loci using MACSE v1.01b.[16] Apart from its primary utility as a codon-aware aligner, MACSE allows the user to align reliable and unreliable protein coding sequences, the latter defined as sequences containing errors like stop codons or frameshift mutations. For each protein coding locus in our dataset, amino acid sequences classified under the Formicidae rank that represented these genes were retrieved from NCBI GenBank Protein database. We reverse transcribed them and passed them to MACSE as reliable input sequence. Then, for each of these loci, a random sample of 50 sequences were passed as unreliable sequences. The resulting multiple sequence alignment generated by MACSE was manually cleaned and curated using Geneious version R8[17] to reduce artifactual gaps. The remaining sequences were then aligned to this multiple sequence alignment using MAFFT v7.205 software[18] with the '–add' and '–keep-length' options. The resulting nine multiple sequence alignments, each containing the full set of sequences, were also manually cleaned to remove poorly aligned or non-coding regions and examined to ensure that a fully codon-based alignment was generated. The two non-coding ribosomal genes, *18S* and *28S*, were aligned with MAFFT and the resulting alignments manually curated. Finally, we concatenated the multiple sequence alignments for all 11 loci into a single matrix containing 679 taxa and 10,767 sites, with 42.2% missing data.

Partitioning Schemes and Model Selection. We used PartitionFinder v1.1.1 software[19] to determine suitable partitioning schemes and corresponding models of molecular evolution for the concatenated multiple sequence alignment generated in the previous step. We designated 29 data blocks: two representing the ribosomal genes *18S* and *28S*, and 27 blocks representing the three codon positions for the nine protein coding genes. Models searched included HKY, HKY+Γ, SYM, SYM+Γ, GTR, GTR+Γ, TrN, TrN+Γ, K80, K80+Γ, TrNef, TrNef+Γ, JC and JC+Γ. We

Supplementary Table 1: Partitions and nucleotide models identified by PartitionFinder v1.1.1[19] from the 11 loci used in the phylogenetic analysis. pos1, pos2 and pos3 refer to codon positions 1, 2 and 3 respectively.

Subset	Best Model	Subset Partitions	Size
p1	TrNef+Γ	18S	1900
p2	GTR+Γ	28S	3062
p3	GTR+Γ	abdA_pos1	211
p4	GTR+Γ	Ubx_pos2, abdA_pos2	428
p5	GTR+Γ	abdA_pos3	211
p6	SYM+Γ	argK_pos1	226
p7	SYM+Γ	argK_pos2	226
p8	TrNef+Γ	Top_pos3, argK_pos3	520
p9	TrNef+Γ	CAD_pos1	372
p10	GTR+Γ	CAD_pos2, Top_pos2	666
p11	TrNef+Γ	CAD_pos3	372
p12	TrN+Γ	EF1aF1_pos1	120
p13	K80+Γ	EF1aF1_pos2	120
p14	SYM+Γ	EF1aF1_pos3	120
p15	SYM+Γ	EF1aF2_pos1	173
p16	GTR+Γ	EF1aF2_pos2	173
p17	SYM+Γ	EF1aF2_pos3	173
p18	SYM+Γ	LwRh_pos1	161
p19	SYM+Γ	LwRh_pos2	161
p20	SYM+Γ	LwRh_pos3	161
p21	SYM+Γ	Top_pos1	294
p22	SYM+Γ	Ubx_pos1	217
p23	K80+Γ	Ubx_pos3	217
p24	TrN+Γ	wg_pos1, wg_pos2	322
p25	GTR+Γ	wg_pos3	161

used the Bayesian Information Criterion option in PartitionFinder for model selection and comparison. PartitionFinder determined 25 subsets from the 29 data blocks. The results are shown in Supplementary Table 1.

We used ClockstaR v2[20] to determine whether multiple unlinked relaxed-clock models were advisable for the partitions determined by PartitionFinder. Based on the "firstSEmax" criterion and the PAM clustering algorithm, multiple ClockstaR runs preferred a single clock model (k=1), so clock models were linked for all relaxed-clock analyses.

Backbone Tree ML Estimation. We reconstructed phylogenetic relationships of 673 ant taxa using the maximum likelihood method of inference implemented in RAxML v8.0.1[21] with the nucleotide alignment generated from data from published sources. We performed 100 ML searches to find the best tree and 1000 bootstraps to obtain support values. The partitioning scheme determined by PartitionFinder was employed with the GTR+Γ model of evolution. This undated backbone tree represents 269 major genera out of 323 described genera, although most of the remaining genera have very few species (98% of ant species are in the included 269 genera).

Backbone Tree Divergence Dating: Overview. The timescale of ant evolution has been a contentious issue in the past[4,5,7] and is relevant for the hypotheses we test in this study. Thus, we performed three dating methods to infer a set of time-calibrated backbone phylogenies. First, we used a traditional approach of calibrating nodes with fossil evidence (henceforth, NC), using and comparing two different choices of prior distributions (uniform and lognormal). Second, we implemented the more recently developed fossilized birth-death process (henceforth, FBD) approach.[22] The FBD approach circumvents problems that arise with node calibration methods and allow the integration of a broader range of fossil data into analyses. However, it may be sensitive to taphonomic biases in the fossil record. In general, each of these approaches has strengths and weaknesses, although the performance of different methods under different conditions is still a point of active research.

We constrained our trees to follow the rooting of the phylogenomic study of Johnson et al. (2013),[23] with ants sister to Apidae and nested within the Aculeata.

Backbone Tree Divergence Dating: Node Calibration (NC) Approach. In this approach, the backbone tree was dated in absolute time using BEAST2.[24] We employed two different methods for the dating process: Yule model of speciation with uniform prior distributions and Yule model of speciation with lognormal prior distributions, each using 51 fossil calibrations. See Supplementary Table 2 for de-

tails regarding these fossil calibrations and their distributions used in BEAST2. The calibration of the root node was set to a uniform prior distribution in both methods reflecting the bounds on that node argued by Brady et al. (2006).[5] For both BEAST2 runs, the partitioning scheme and models specified by PartitionFinder were used (Supplementary Table 1), along with a single (linked) Lognormal Relaxed-Clock model (based on the ClockstaR analysis).

Supplementary Table 2: The fossil calibrations used for the Node Calibration dating method, and the parameters of the prior distributions (in million years) used assuming uniform or lognormal prior distributions in BEAST2. Calibrations were consolidated from Brady et al. 2006,[5] Moreau et al. 2006,[4] Ward et al. 2010,[6] Schmidt 2013,[13] Brady et al. 2014,[14] Ward et al. 2015.[8] UL = Uniform Prior Lower Bound; UU = Uniform Prior Upper Bound; LO = Lognormal Prior Offset; LM = Lognormal Prior Median; LU = Lognormal Prior Upper 95%.

Reference	Stem Lineage	UL	UU	LO	LM	LU
Mackay 1996;[25] De Andrade 1998[26]	*Acanthostichus*	15	140	15	44	100
Arillo & Ortuño 2005[27]	*Acropyga*	15	140	15	44	100
De Andrade 1994[28]	*Anochetus*	15	140	15	44	100
Schultz 2007[29]	*Apterostigma*	15	140	15	44	100
Wilson 1985;[30] Wild & Cuezzo 2006[31]	MRCA *Azteca* and *Gracilidris*	15	140	15	44	100
De Andrade & Baroni Urbani 1999[32]	*Cephalotes atratrus*	15	140	15	44	100
Blaimer 2012[33]	*Crematogaster acuta*	15	140	15	44	100
Baroni Urbani & Wilson 1987[34]	*Leptomyrmex* stem	15	140	15	44	100
Wilson 1985[30]	*Neivamyrmex*	15	140	15	44	100
De Andrade et al. 1999[35]	*Nesomyrmex* (*wilda* + *echatinodis*)	15	140	15	44	100
Wilson 1988[36]	*Octostruma*	15	140	15	44	100
Wilson 1985b[37]	*Prionopelta*	15	140	15	44	100
Ward 1992[38]	*Pseudomyrmex*	15	140	15	44	100
Wilson 1988[36]	*Solenopsis* (*fugax* + *xyloni*)	15	140	15	44	100
						Continued ...

Supplementary Table 2 (continued)

Reference	Stem Lineage	UL	UU	LO	LM	LU
Baroni Urbani & De Andrade 1994,[39] 2007[40]	*Strumigenys ambatrix*	15	140	15	44	100
Dlussky & Rasnitsyn 2009[41]	*Temnothorax poeyi*	15	140	15	44	100
Baroni Urbani 1980[42]	*Trachymyrmex*	15	140	15	44	100
De Andrade 1998[26]	*Discothyrea*	22.5	140	22.5	48	100
Emery 1891[43]	*Cataulacus*	30	140	30	53	100
Bolton 2007[44]	*Technomyrmex* stem	30	140	30	53	100
Carpenter 1930[45]	*Pogonomyrmex* (nearctic clade and *Hylomyrma* MRCA)	34	140	34	56	100
Dlussky 2009[46]	*Amblyoponinae*	42	140	42	61	100
Dlussky 1997[47]	*Anonychomyrma*	42	140	42	61	100
Wheeler 1915[48]	*Aphaenogaster* + *Goniomma*	42	140	42	61	100
Dlussky 1997[47]	*Camponotus*	42	140	42	61	100
Mayr 1868;[49] Wheeler 1915[48]	*Carebara*	42	140	42	61	100
Dlussky 2002[50]	MRCA *Dolichoderus pustulatus* and *decollatus*	42	140	42	61	100
Dlussky 1997[47]	*Formica*	42	140	42	61	100
Dlussky 1997[47]	*Hypoponera*	42	140	42	61	100
Dlussky 2011[51]	MRCA *Lasius*, *Myrmecocystus*, *Nylanderia*	42	140	42	61	100
Dlussky & Rasnitsyn 2009[41]	*Myrmica*	42	140	42	61	100
Wheeler 1915[48]	*Ponerinae*	42	140	42	61	100
Dlussky & Radchenko 2011[52]	*Pristomyrmex*	42	140	42	61	100
De Andrade & Baroni Urbani 2003[53]	*Proceratium*	42	140	42	61	100
						Continued ...

Supplementary Table 2 (continued)

Reference	Stem Lineage	UL	UU	LO	LM	LU
Dlussky 1997[47]	*Rhytidoponera*	42	140	42	61	100
Branstetter 2012[54]	*Stenamma*	42	140	42	61	100
Wheeler 1915;[48] Dlussky & Rasnitsyn 2009[41]	*Tatuidris*	42	140	42	61	100
Dlussky & Rasnitsyn 2009[41]	*Temnothorax*	42	140	42	61	100
Dlussky 1997[47]	*Tetramorium*	42	140	42	61	100
Dlussky 1997[47]	*Tetraponera*	42	140	42	61	100
Poinar et al. 1999;[55] Moreau et al 2006[4]	Tapinomini crown	55	140	55	76	120
Engel & Grimaldi 2005;[56] Dlussky 2009[41]	*Ectatomminae*	79	140	79	92	120
Darling & Sharkey 1990;[57] Grimaldi & Engel 2005[58]	*Aglyptacros*	90	185	90	121	185
Grimaldi & Agosti 2000[59]	Formicinae	92	140	92	108	140
Engel & Grimaldi 2005;[58] Dlussky 1999[60]	Dolichoderinae	100	140	100	120	140
Grimaldi & Engel 2005[58]	*Aporus*	100	185	100	128	185
Antropov 2000[61]	*Chalybion*	100	185	100	128	185
Zhang et al. 2002;[62] Rasnitsyn et al. 1998[63]	*Apis* + *Chalybion*	140	185	140	155	185
Brady et al. 2006[5]	ROOT: MRCA Aculeata	145	185	uniform prior used in both analyses		

For both dating methods we sought to use reasonable starting trees to reduce burnin time. We generated these by dating the ML backbone tree using the 51 age calibrations with *chronos* function provided in APE package[64] in R. The starting trees satisfied the monophyly constraints enforced by the fossil calibrations and were fixed (i.e. we did not estimate tree topology with BEAST2). Four independent analyses were performed for each of the two methods. Each analysis was monitored

using Tracer software v1.6.0[65] to evaluate burnin. We wrote a custom script to monitor convergence of node-age estimates across runs (producing log files readable in Tracer), which occurred as the runs ranged from 129 million states to 141 million states. We discarded the leading 40% of the saved states, and combined the trees from the different runs into a single posterior sample.

Backbone Tree Divergence Dating: Fossilized Birth-Death Process (FBD) Approach. We first generated a list of candidate ant fossil taxa from a recently consolidated global dataset of ant fossils.[66] The FBD analysis is implemented by specifying a tip date for all taxa (extant taxa have a tip date of zero, whereas fossils appear on the phylogeny at some past date), and a set of monophyly constraints which constrain fossils to fall within particular clades (thereby affecting the age of those clades). Here, we did not use ML backbone tree directly in the analysis, but instead used the backbone tree and previous work on ant phylogenetics to justify 217 monophyly constraints reflecting well-supported relationships. These constraints included 25 subfamily level constraints, 177 genus level constraints, and 15 constraints on groups of species that are monophyletic but within non-monophyletic genera (see Supplementary Data 2). Genera which have not been recovered as monophyletic in the ML tree and previous studies (e.g. *Monomorium* and *Cerapachys*) were not constrained to be monophyletic. We relied on taxonomy as a guide to placing fossils into clades. For example, an extinct genus that is classified in the subfamily Myrmicinae is constrained to fall within the myrmicine clade, however, it would be excluded from falling within individual genera by genus-level monophyly constraints. We excluded any fossils that are *incertae sedis* or were identified as falling in a non-monophyletic genus. A list of fossil taxa used and their tip dates can be found in Supplementary Data 1.

We used the Sampled Ancestors plugin[67] in BEAST2[24] to implement this analysis. Combined with fossil taxa tip dates, the Sampled Ancestors package automatically generates starting trees using these constraints. These trees, however, had either extremely short or extremely long internal branch lengths, which led to undesirably long burnin times on this large a tree. To reduce the burnin, we manually adjusted these branches (without changing fossil tip dates) to give them more realistic lengths and in this way generated four starting trees. Unlike the NC approach, in which topology was fixed to the ML topology, the FBD analysis integrates over tree space consistent the monophyly constraints. For each start tree, we ran four independent analyses, making a total of 16 analyses. As before, the runs were monitored with Tracer software using a script to evaluate node age convergence (of post-burnin states) across runs. We stopped the runs when they were ranging from 170 million

states to 229 million states. We discarded the first 40% of the states, and combined the runs into a single posterior sample.

Backbone Tree Divergence Dating: Method Comparison. The FBD and NC approaches both have their strengths and weaknesses, and a full evaluation of those is best explored elsewhere. We compared the recovered node ages found using the different dating methods, by comparing median ages of a set of 279 nodes present in all trees. We found that the two Node-Calibration approaches (lognormal and uniform priors) gave very similar results, but the FBD was more divergent from the other two, although still highly correlated (Supplementary Fig. 8). Part of the reason could be that the two Node-Calibration approaches were based on the same (ML) topology, while the FBD analysis sampled over the topology posterior as well as node ages. Given the insensitivity of the NC approach to the choice of calibration prior, from this point we used trees dated with FBD and only one of the NC methods: the version based on less restrictive uniform prior calibrations.

Clade Grafting and All-Ant Trees. We sought to use the posterior trees from the Bayesian divergence dating analyses to generate trees representing the full range of known taxonomic diversity in the Formicidae family. This follows similar efforts for plants,[1] birds,[2] and mammals.[3] Those comprehensive phylogenies, while not fully resolved or based on molecular data for all taxa, have proven to be very useful for evolutionary biology and applications in ecology (e.g. community phylogenetics). Note that we also use backbone trees for certain downstream analyses when all-ant trees were not appropriate or to corroborate results.

Of the 14,912 taxa in our geographic dataset, 14,594 can be placed onto the phylogeny. The remainder belong to (often very small) genera for which no molecular data are available. To place taxa onto the backbone trees, we identified 262 terminal ant clades on the backbone tree (Supplementary Data 3). These terminal clades mostly corresponded to genus names. However, in cases of non-monophyletic genera, the terminal clades were composed of either sub-genus or multi-genus clades. If a genus was split into two terminal clades, we assigned species randomly to the two clades in proportion to the number of taxa in each clade on the backbone tree (but in this case which species was assigned to each clade varied ranodmly across each all-ant tree we constructed). For each of these sets of species, we generated 2000 pure-birth trees with a matching richness using the *pbtree* function provided in the phytools package[68] in R. Then, we randomly retrieved 800 dated backbone trees from the posterior set of each of the two dating methods, NC and FBD, - 1,600 trees in total. On each of the backbone trees, we replaced the 262 terminal clades (having

incomplete species composition) with random trees, without replacement, from the respective pure-birth tree sets (having near complete species composition).

The key decision in grafting the terminal clades onto the backbone is how to set the crown age of the grafted clade. The backbone trees usually contain more than one specimen from the same terminal clade. This gives a lower-bound on the crown age of the clade, whereas the upper bound is given by the stem node. In many cases, the crown node on the backbone is likely the true crown node of the clade, for others it is almost certainly not. Assuming the crown nodes on the backbone are the true nodes can underestimate clade age and overestimate diversification rate. Assuming the true crown is older (located somewhere between the backbone crown age and stem age) may lead to overestimating ages and underestimating diversification rates. Because neither solution is perfect, we performed the analyses using both approaches and compared results for those analyses that used these ant-wide trees.

We call the two approaches the "stem grafting" and "crown grafting" methods. For the stem grafting method, to make a single all-ant tree we 1) first chose a backbone tree from the posterior, then 2) for each terminal clade, we found the crown and stem node for that clade on the backbone tree, 3) we set the crown of the terminal clade to a randomly chosen age between the backbone crown and stem ages, and scaled the edge lengths of the grafted clade using the randomly chosen age. For the crown grafting method, we followed the same procedure except we set the crown age of the grafted clade to match the crown age on the backbone tree, and scaled the edge lengths using this age. In both approaches, if only one taxon appears on the backbone tree (i.e. thus it is a single branch there is no crown on the backbone), we grafted the crown of the clade to a random age between the stem node and the present.

In total, the above procedures produced four sets of 800 all-ant trees, each with 14,594 species, constructed with combinations of the two dating methods (NC and FBD) and the two grafting methods ("stem grafting" and "clade grafting"). Individual trees will contain artifactual variation due to the random structure within terminal clades, and reflect individual samples from the posterior of backbone node ages, but collectively the tree sets integrate over these variations.

We additionally created a single tree for each method where we first set the ages of the backbone tree to their median values across the backbone posterior tree set (for FBD trees, where topology changes as well, we used the maximum clade credibility topology). These trees were used to give a single "average" tree for each method (e.g. for visualization purposes), and for the sampling bias tests, but were not used for the main statistical analyses and conclusions. We refer to these as the "mcc" trees.

Supplementary Note 2: Diversification Rate Inference and Tests for Correlations with Latitude

Overview. Statistical inference of macroevolutionary rates from phylogenetic data is a fundamental, but technically challenging task in evolutionary biology. New methods are actively being developed, however these are controversial with the performance of different methods under debate. Ideally, one would like to infer speciation rates, extinction rates, and net diversification rates in a way that is robust to lineage-specific heterogeneity in those rates and accounts for missing data across lineages. However, this has proven to be a challenging task. For example, some previous studies on the latitudinal gradient have used methods to detect latitudinal dependency that are prone to false inference when there is background lineage-specific heterogeneity in macroevolutionary rates, which always be the case for a tree as large as an entire group like ants (or birds, mammals, etc.)[69] Likewise, methods such as MEDUSA[70] that infer heterogeneity in clade rates and are intended to account for missing data can also be problematic.[71] We designed our analyses around BAMM (Bayesian Analysis of Macroevolutionary Mixtures),[72] but very recently this method has been under debate[73,74] as well (see additional discussion below).

In this context, we took a conservative approach in what we sought to infer, while foregoing opportunities to infer some macroevolutionary rates that we might ideally like to know. Specifically, we focused on net diversification rates, the difference between speciation and extinction rates, rather than inferring variation in both speciation and extinction across the tree. The reason we chose to do this is that teasing apart speciation and extinction rates is more challenging than inferring net diversification rate, and the former depends strongly on the details of branching patterns within clades in the tree. Tree shape, in turn, can be affected by taxa not included in the tree, and for a group as large as ants (including birds and mammals) no phylogeny is completely sampled with molecular data for all species. While methods exist that attempt to account for lineage specific sampling, the validity of these has recently been questioned.[71]

We thus used two alternate methods for representing unsampled species on the tree. First, we ran BAMM on the backbone trees, assigning sampling fractions to each terminal clade based on known richness of the clade. Second, we used the completely sampled trees (with respect to known ant species) that were constructed with the grafting methods described above, but without clade-specific sampling fractions. The species assigned to each clade reflect well-supported relationships in ant systematics, but the internal branching pattern is generated with a pure birth process. This should allow for inference of overall net diversification rates but would give problem-

atic estimates of clade-specific speciation and extinction rates, thus we ignored the latter. For similar reasons, we focused on average net diversification rate of different lineages and clades by assuming constant diversification rate models rather than those that allow for density-dependence (accelerating or decelerating diversification within clades), because the latter would be biased by the grafting approach.

This focus on net diversification rate is sufficient to test the hypothesis that variation in macroevolutionary rates is responsible for the disparity of richness across latitude. Although teasing apart any variation in speciation and extinction with latitude would be interesting for many reasons, if systematic variation in those rates does not lead to variation in net diversification rate with latitude, then variation in those rates cannot be a causal factor in the latitudinal diversity gradient.

Bayesian Inference of Diversification Rates. To infer net diversification across ant clades, we used BAMM v2.5.0.[72] We randomly selected 100 trees from each of the four groups of all-ant trees and ran BAMM on each tree. We ran between 18 and 20 million generations using MEDUSA-like settings (enforcing constant rate birth-death) in BAMM, saving 1000 states per tree. Speciation/Extinction priors were set using BAMMtools[75] function, *setBAMMpriors*, for each of the 400 trees. We set *expectedNumberOfShifts* to 250, and *minCladeSizeForShift* to 5. We discarded 50% of the states as burn-in, and used the posterior set for summarizing the net diversification rates of the 262 terminal clades on each tree. We also performed tests to ensure that the results were not prior-sensitive, by altering the prior and examining the number of rate-shifts.

We also ran BAMM on the backbone trees (100 each from NC and FBD dating methods) using the sampling fraction option to represent missing species. Here we set the sampling fraction for each tip to be the number of species on the backbone phylogeny for each clade divided by the total known richness of that clade. For these runs, we set the *expectedNumberOfShifts* to 1 and *minCladeSizeForShift* to 1. The BAMM control files can be found in the Dryad repository (doi:10.5061/dryad.g579t7k).

Cross-validation of BAMM results. BAMM has very recently been debated on both theoretical and practical grounds,[73,74] and it is likely this debate will continue for some time. To our knowledge, there is no alternative, uncontroversial method that infers heterogeneity in diversification rates across large trees with many potential rate-shifts. In light of these developments, it is worth questioning whether our BAMM results are reasonable or artifactual in some way due to the performance of the program.

As described above, we intentionally took a conservative track in what we hoped

to achieve with our diversification analysis. We did not seek to infer speciation and extinction rates individually, only net diversification rates, we did not rely solely on corrections that adjust for "incomplete sampling fractions" (i.e. we also constructed all-ant trees), we only considered constant-rate models (i.e. no accelerating or decelerating models), and we did not focus on the number and location of individual rate shifts. In this context, diversification rates should be mostly be a function of clade age and richness. BAMM provides a convenient framework to infer this across entire trees, integrate over uncertainty, and allow for sharing of information across individual clades that are close in the phylogeny. But does it give reasonable results that are driven by clade ages and richness? To check this, we compared the net diversification rate parameter inferred with BAMM (median from posterior) with that estimated from *bd.ms* function in geiger v2.0[76] that fits a single constant-rate model that depends only on clade age and richness. We used the all-ant trees derived from the mcc-median trees from the four methods NC-stem, NC-crown, FBD-stem, FBD-crown. Parameters *epsilon* and *missing* in *bd.ms* were set to 0.

On a species level the correlation between the results from BAMM and geiger's *bd.ms* function is very high (Spearman's ρ: 0.93, 0.90, 0.93, 0.94, for NC-crown, NC-stem, FBD-stem, and FBD-crown respectively). We also compared estimates on a clade-by-clade basis, excluding very small clades (<20 species) which should have uncertain estimates with any method. On this clade-by-clade basis, the results are highly correlated (Spearman's ρ: 0.94, 0.89, 0.88, 0.94, for NC-crown, NC-stem, FBD-stem, and FBD-crown respectively). Thus, while we cannot comment on the validity of BAMM for all purposes, we believe it represents a valid approach that gives reasonable values for the quantities we want to infer, and our overall conclusions would be similar using different approaches.

Testing for latitude-diversification rate correlations. After estimating diversification rates with BAMM, we took two complementary methodological approaches to evaluating correlations between diversification rate and latitude. We used multiple methods in order to ensure our results were robust to variations in data coding and methodology. First, we performed clade-wise regression approach in a phylogenetic framework. Second, we used lineage-wise approach based on structured rate permutations.

Clade-wise weighted PGLS approach. We performed a series of clade-wise regressions to evaluate if there was any correlation between the latitudinal affinity of a clade and its inferred diversification rate. First, as a baseline, we calculated an unweighted correlation between clade diversification rate and the average of ab-

solute midpoint latitudes from all species in the clade, and calculated this across all trees from the posterior sample (GLS). Second, to correct for phylogenetic non-independence among clades, we performed regressions weighted by phylogeny using a PGLS regression approach.[77] To represent phylogenetic covariation across trees, for each posterior tree we pruned each terminal clade producing new termini located at the crown node of each clade.

Third, given that our estimates of clade age and thus diversification rate have uncertainty, we weighted the regression to compensate for this uncertainty (wGLS and wPGLS). Specifically, for the regression on each tree, we weighted data points by the inverse coefficient of variation for the clade's diversification rate estimate across all trees. We also weighted each clade by the confidence intervals on its average absolute latitude. Thus, clades that have more uncertainty in their diversification rate (for example due to uncertainty in clade age in the backbone or arising from the grafting process), or are more spread across latitude, contribute less to the regression than those clades that have more confidently-estimated rates or are more concentrated at a given latitude.

For all regressions, we scaled predictor and response variables by their standard deviation and centered their distribution on zero so as to produce a standardized regression coefficient. To assess the relative effects of phylogenetic correction and weighting on model parameters, we repeated these analyses for each combination of phylogenetically corrected and un-corrected, weighted and un-weighted GLS settings.

We performed these regressions on BAMM results using both the clade grafting method (i.e. adding species to the tree) and also on the BAMM results from the backbone trees using the sampling fraction option to represent missing species. For each terminal clade, we exported the average net diversification rate of the clade since the crown node. If there was only one species in the clade, we took the average rate along the branch from the stem node.

Lineage-wise permutation test approach (STRAPP). As a complementary test, we used the *traitDependentBAMM* function, also referred to as STRAPP - Structured Rate Permutations on Phylogenies,[78] in the BAMMtools[75] R package. This function calculates a correlation coefficient and tests for significance by permuting diversification rates on the phylogeny while preserving the underlying covariation in macroevolutionary rates and species traits.

We ran STRAPP on each posterior tree: 100 each from the four groups of all-ant grafted trees (NC-stem, NC-crown, FBD-stem, FBD-crown) and 100 each of the two groups of backbone/sampling-fraction trees (NC and FBD). For the all-ant trees, we used the species level midpoint latitudes. For the backbone trees, we assigned each

clade the average absolute midpoint latitude for all species in the whole terminal clade (reasoning that sampling fraction is being assigned based on whole clade richness, thus whole-clade tropical affinity should be reflected). We also tested using individual species midpoint latitudes using only those taxa found on the backbone tree, and found no difference in results. We tested statistically whether there was a significant correlation between absolute midpoint latitude and diversification rate using the Pearson correlation as a test statistic and 1000 null replications.

Supplementary Note 3: Evaluating potential effects of sampling bias on results

Our analysis is near-comprehensive with respect to current knowledge of ant species and their geographic distributions, but the global inventory of ant biodiversity is far from complete. Furthermore, it is probable that tropical fauna are not as well documented as extratropical fauna. Here we consider how the three main analyses presented in this paper, phylogenetic diversity, and ancestral states/lineages through time, and diversification rates, might or might not be biased by latitudinal sampling given our results.

Phylogenetic diversity and latitudinal sampling bias. For phylogenetic diversity, we found that extratropical species were clustered on the phylogeny, especially in the northern hemisphere (Figs. 1, 2, Supplementary Fig. 2). Overall, MPD should be rather insensitive to sampling bias because it reflects the overall spread of species in a sample across the tree. If new species are added to the tropical clades more or less in the same proportions as their current richness (a reasonable assumption), the MPD in the tropics should be stable. We found phylogenetic diversity to be lower in the extratropics because lineages there are clustered into relatively few clades. If we sampled more species from the tropics, that would not make the extratropics less clustered on the phylogeny. This may not be true for all PD measures, for example mean nearest taxon distance (MNTD) would be sensitive to density of species within clades rather than spread among them.

Diversification rate and latitudinal sampling bias. In principle, the undersampling of the tropics could bias our estimate of diversification rates and obscure potential latitudinal correlations. To investigate the likelihood of this possibility, we

performed experiments to mimic potential latitudinal sampling biases and evaluate their effects on our results using two methods.

In the first method (Test 1), we used the grafted "all-ant" trees and randomly pruned different fractions of extratropical tips from the tree and re-ran BAMM. This is intended to account for a lower probability of discovery of tropical species relative to extratropical species (i.e. by undiscovering extratropical species) without using sampling fractions. Here we randomly pruned off extratropical species with a range of probabilities $(0.2, 0.4, 0.6)$. The maximum value (0.6) would imply that even if extratropical species are completely sampled, tropical species are only 40% sampled. Since there are over 10,000 described ant species in the tropics, this would mean there are actually 25,000 tropical species and thus close to 30,000 ant species total, which is at the high end for estimates of total ant species.[79] We performed two replicates for each tree/parameter combination, but since the results were similar across replicates, for clarity of presentation we only present one replicate.

In the second test (Test 2), we used the backbone trees and the clade-specific sampling fraction option in BAMM, and manipulated the sampling fractions to mimic undersampling of tropical taxa. There are two uncertainties we need to account for: the number of total remaining ant species and the fraction of those that are tropical. For total ant species, the current number is approximately 15,000. We estimate the true number to be within the range of 20,000-30,000. For the fraction tropical (i.e. absolute midpoint latitude < 23.5), the current number is approximately 0.7, which should provide a minimum value, thus we estimate the fraction of remaining species to be between $0.7-1.0$, with 1.0 reflecting the extreme assumption that all undiscovered ant species are tropical. We used these numbers to adjust the clade-specific sampling fractions in BAMM. We assumed that remaining tropical and temperate species are assigned to clades in the same proportions of current species. For example, if 1% of all known tropical ant species are found in clade A, then we assume 1% of undiscovered tropical ant species are also in that clade. Thus, using the ranges above, we first calculated how many tropical and extratropical species are missing from the tree, and then how many should be assigned to each clade. Finally, we adjust the sampling fractions accordingly and re-ran BAMM.

Both the analyses above were performed on the mcc trees for each dating method. After inferring the new rates in BAMM, we put the results through the same analytical pipeline for testing diversification rate-latitude correlations. For test 1 (pruning method) we recalculated the average absolute latitude for each clade after dropping tips. For the sampling fraction test (test 2), we performed the PGLS and STRAPP analyses with a new independent variable: the fraction of species in the clade that are tropical (according to the binary classification). We used the latter so we can

account for the fact that if sampling is biased by latitude, it also will affect the clade-level average tropicality. In other words, we can adjust the fraction tropical in each clade more straightforwardly than the average latitude in each clade, because the latter requires a model of specifically how bias changes with latitude. We also, however, ran the regressions on the original average absolute latitude variable and the results were comparable.

Both of our tests agree that it is unlikely that latitudinal sampling bias is obscuring a diversification rate-latitude correlation (Supplementary Fig. 5). Even making extreme assumptions about latitudinal sampling bias (there are 15,000 ant species out there undiscovered, and they are all in the tropics), the correlation goes from weakly positive to near zero, and never becomes significantly negative. We believe this is due to the extreme heterogeneity in richness and diversification rates among ant clades at all latitudes, which assuming that missing species are distributed in proportion to current richness, still dominates the variation in diversification rate even if accounting for a latitudinal sampling bias.

Ancestral state estimation and sampling bias. For reconstructing ancestral states/lineages through time, having more species on the phylogeny would increase the accuracy of those reconstructions, but would a latitudinal sampling bias systematically skew the results? Our main finding was that there are many old tropical lineages relative to few old extratropical lineages. If we oversampled extratropical lineages, this could only increase the relative number of old extratropical lineages relative to tropical lineages. In addition, tropical undersampling could bias the extratropical lineages to look older than they are, because we might miss recent tropical relatives. Thus, if there is a bias, we believe it should be in the opposite direction of our results.

A related concern is sample size itself. Our ancestral state estimation uses the backbone trees constructed with molecular data. Out of total 679 taxa on the backbone trees, 512 have species range data (i.e. because they are described species) and can be used for ancestral state reconstruction. Ancestral state reconstruction does not require a fully sampled phylogeny and should not be biased by using random subsets of taxa. However, it is reasonable to question whether our ancestral state results are sensitive to sample size or inclusion of particular taxa, given that the actual taxa on the tree are far fewer than the total number of ant species. To check this, we performed two simple subsampling tests. First, in test 1 we randomly chose 25% of the taxa, and dropped this same set of taxa from all the trees. Second, in test 2 we dropped a different randomly chosen 25% of taxa from each posterior tree. We then recalculated the ancestral state pattern across trees as we did for the full

dataset (Figure 3, Supplementary Figs. 6-7). We performed these tests using the time-homogeneous asymmetric model, using NC backbone trees.

We found that the overall ancestral state pattern, showing old tropical lineages but few or no old temperate lineages, was highly stable even after dropping 25% of the taxa in both tests (Supplementary Fig. 10). This indicates that the pattern we recovered is not sensitive to sample size or the particular choice of taxa included.

Supplementary Note 4: Additional discussion of ancestral state estimation results

We successfully fit the time-homogeneous symmetric and asymmetric models (Figure 3, Supplementary Fig. 6), and the 2-epoch symmetric model (Supplementary Fig. 7). We also attempted to fit 2-epoch asymmetric models, but found they exhibited convergence issues and inconsistent results. We believe these problems arise from fitting asymmetric models to scenarios of accelerating evolution (scenarios involving decelerating evolution can more easily be fit successfully, see ref.[80]). For example, a pattern where old lineages are mostly in one of the two states (which appears to be our case) could be caused by either very low evolutionary rates and an ancestor in that state, or very fast but very asymmetric evolution, causing most lineages to accumulate in one state. Thus, the recent emergence of a minority trait can be caused by either an acceleration or deceleration in evolutionary rate with a similar resulting pattern. Due to these issues we do not further consider 2-epoch asymmetric model in this study.

The time-homogenous asymmetric model (FBD: $\overline{\Delta \text{AIC}} = 0.04 \pm 0.004$, NC: $\overline{\Delta \text{AIC}} = 0.005 \pm 0.004$, with the means and standard errors taken across posterior trees) and the symmetric epoch-model (FBD: $\overline{\Delta \text{AIC}} = 2.5 \pm 0.14$, NC: $\overline{\Delta \text{AIC}} = 3.3 \pm 0.12$) fit the data comparably well, and both outcompeted the time-homogeneous symmetric model (FBD: $\overline{\Delta \text{AIC}} = 12.0 \pm 0.16$, NC: $\overline{\Delta \text{AIC}} = 16.7 \pm 0.17$). Since the asymmetric model had the lowest AIC for most trees, we presented it in the main text. Both models predict a recent emergence of extratropical lineages, but for somewhat different reasons. The symmetric-epoch models predict this is due to there being a tropical ancestor and acceleration of transition rates in the recent past. The time-homogenous asymmetric predict this due to a sustained 3-fold higher transition rate from the extratropics back into the tropics, resulting in few old extratropical lineages persisting. For the epoch model, the optimal breakpoint was actually more

recent than 34mya Oligocene cooling, falling during the Miocene (NC mean time shift: 19.5 ± 0.65mya, FBD mean time shift: 16.9 ± 0.3mya). However, the likelihood of an Oligocene breakpoint is not dramatically different (Supplementary Fig. 7). Further work is needed to distinguish strongly asymmetric evolution versus more symmetric, but time-inhomogeneous trait evolution.

Supplementary References

[1] Webb, C. O. and Donoghue, M. J. Phylomatic: tree assembly for applied phylogenetics. *Molecular Ecology Notes*, **5**, 181–183, (2005).

[2] Jetz, W., Thomas, G. H., Joy, J. B., Hartmann, K., and Mooers, A. O. The global diversity of birds in space and time. *Nature*, **491**, 444–448, (2012).

[3] Kuhn, T. S., Mooers, A. Ø., and Thomas, G. H. A simple polytomy resolver for dated phylogenies. *Methods in Ecology and Evolution*, **2**, 427–436, (2011).

[4] Moreau, C. S., Bell, C. D., Vila, R., Archibald, S. B., and Pierce, N. E. Phylogeny of the ants: diversification in the age of Angiosperms. *Science*, **312**, 97–101, (2006).

[5] Brady, S. G., Schultz, T. R., Fisher, B. L., and Ward, P. S. Evaluating alternative hypotheses for the early evolution and diversification of ants. *Proceedings of the National Academy of Sciences*, **103**, 18172–18177, (2006).

[6] Ward, P. S., Brady, S. G., Fisher, B. L., and Schultz, T. R. Phylogeny and biogeography of Dolichoderine ants: effects of data partitioning and relict taxa on historical inference. *Systematic Biology*, **59**, 342–362, (2010).

[7] Moreau, C. S. and Bell, C. D. Testing the museum versus cradle tropical biological diversity hypothesis: phylogeny, diversification, and ancestral biogeographic range evolution of the ants. *Evolution*, **67**, 2240–2257, (2013).

[8] Ward, P. S., Brady, S. G., Fisher, B. L., and Schultz, T. R. The evolution of myrmicine ants: phylogeny and biogeography of a hyperdiverse ant clade (Hymenoptera: Formicidae). *Systematic Entomology*, **40**, 61–81, (2015).

[9] Sanderson, M., Donoghue, M., Piel, W., and Eriksson, T. TreeBASE: a prototype database of phylogenetic analyses and an interactive tool for browsing the phylogeny of life. *American Journal of Botany*, **81**, 183, (1994).

[10] Benson, D. A., Cavanaugh, M., Clark, K., Karsch-Mizrachi, I., Lipman, D. J., Ostell, J., and Sayers, E. W. GenBank. *Nucleic Acids Research*, **41**, D36–D42, (2013).

[11] Schultz, T. R. and Brady, S. G. Major evolutionary transitions in ant agriculture. *Proceedings of the National Academy of Sciences*, **105**, 5435–5440, (2008).

[12] Lapolla, J. S., Brady, S. G., and Shattuck, S. O. Phylogeny and taxonomy of the *Prenolepis* genus-group of ants (Hymenoptera: Formicidae) *Systematic Entomology*, **35**, 118–131, (2010).

[13] Schmidt, C. Molecular phylogenetics of ponerine ants (Hymenoptera: Formicidae: Ponerinae). *Zootaxa*, **3647**, 201, (2013).

[14] Brady, S. G., Fisher, B. L., Schultz, T. R., and Ward, P. S. The rise of army ants and their relatives: diversification of specialized predatory doryline ants. *BMC Evolutionary Biology*, **14**, 93, (2014).

[15] Bolton, B. *AntCat: An online catalog of the ants of the world.* (2012).

[16] Ranwez, V., Harispe, S., Delsuc, F., and Douzery, E. J. P. MACSE: Multiple Alignment of Coding SEquences Accounting for Frameshifts and Stop Codons. *PLoS ONE*, **6**, e22594, (2011).

[17] Kearse, M., Moir, R., Wilson, A., Stones-Havas, S., Cheung, M., Sturrock, S., Buxton, S., Cooper, A., Markowitz, S., Duran, C., Thierer, T., Ashton, B., Meintjes, P., and Drummond, A. Geneious Basic: An integrated and extendable desktop software platform for the organization and analysis of sequence data. *Bioinformatics*, **28**, 1647–1649, (2012).

[18] Katoh, K. and Standley, D. M. MAFFT Multiple Sequence Alignment Software Version 7: Improvements in performance and usability. *Molecular Biology and Evolution*, **30**, 772–780, (2013).

[19] Lanfear, R., Calcott, B., Ho, S. Y. W., and Guindon, S. PartitionFinder: Combined selection of partitioning schemes and substitution models for phylogenetic analyses. *Molecular Biology and Evolution*, **29**, 1695–1701, (2012).

[20] Duchêne, S., Molak, M., and Ho, S. Y. W. ClockstaR: choosing the number of relaxed-clock models in molecular phylogenetic analysis. *Bioinformatics*, **30**, 1017–1019, (2014).

[21] Stamatakis, A. RAxML version 8: a tool for phylogenetic analysis and post-analysis of large phylogenies. *Bioinformatics (Oxford, England)*, **30**, 1312–1313, (2014).

[22] Heath, T. A., Huelsenbeck, J. P., and Stadler, T. The fossilized birth-death process for coherent calibration of divergence-time estimates. *Proceedings of the National Academy of Sciences*, **111**, E2957–E2966, (2014).

[23] Johnson, B., Borowiec, M., Chiu, J., Lee, E., Atallah, J., and Ward, P. Phylogenomics resolves evolutionary relationships among ants, bees, and wasps. *Current Biology*, **23**, 2058–2062, (2013).

[24] Bouckaert, R., Heled, J., Kühnert, D., Vaughan, T., Wu, C.-H., Xie, D., Suchard, M. A., Rambaut, A., and Drummond, A. J. BEAST 2: A Software Platform for Bayesian Evolutionary Analysis. *PLoS Comput Biol*, **10**, e1003537, (2014).

[25] MacKay, W. P. A revision of the ant genus *Acanthostichus* (Hymenoptera: Formicidae). *Sociobiology*, **27**, 129–179, (1996).

[26] De Andrade, M. L. First description of fossil *Acanthostichus* from Dominican amber (Hymenoptera: Formicidae). *Mitteilungen der Schweizerischen Entomologischen Gesellschaft*, **71**, 269–274, (1998).

[27] Arillo, A. and Ortuño, V. M. Catalogue of fossil insect species described from Dominican amber (Miocene). *Stuttgarter Beitrage zur Naturkunde. Serie B (Geologie und Palaontologie)*, **352**, 1–68, (2005).

[28] De Andrade, M. L. Fossil Odontomachini ants from the Dominican Republic.(Amber Collection Stuttgart: Hymenoptera, Formicidae. VII: Odontomachiti). *Stuttgarter Beitrage zur Naturkunde (B)*, **199**, 1–28, (1994).

[29] Schultz, T. R. The fungus-growing ant genus *Apterostigma* in Dominican amber. *Memoirs of the American Entomological Institute*, **80**, 425–436, (2007).

[30] Wilson, E. O. Ants of the Dominican Amber (Hymenoptera: Formicidae). 3. The subfamily Dolichoderinae. *Psyche*, **92**, 17–38, (1985).

[31] Wild, A. L. and Cuezzo, F. Rediscovery of a fossil dolichoderine ant lineage (Hymenoptera: Formicidae: Dolichoderinae) and a description of a new genus from South America. *Zootaxa*, **1142**, 57–68, (2006).

[32] De Andrade, M. L. and Baroni Urbani, C. Diversity and adaptation in the ant genus *Cephalotes*, past and present. *Stuttgarter Beitrage zur Naturkunde (B)*, **271**, 1–889, (1999).

[33] Blaimer, B. Acrobat ants go global–origin, evolution and systematics of the genus *Crematogaster* (Hymenoptera: Formicidae). *Molecular Phylogenetics and Evolution*, **65**, 421–436, (2012).

[34] Baroni Urbani, C. and Wilson, E. O. The fossil members of the ant tribe Leptomyrmicini (Hymenoptera: Formicidae). *Psyche*, **94**, 1–8, (1987).

[35] De Andrade, M. L., Baroni Urbani, C., Brandao, C. R. F., and Wagensberg, J. Two new species of *Leptothorax* "*Nesomyrmex*" fossils in Dominican amber (Hymenoptera: Formicidae). *Beitrage zur Entomologie*, **49**, 133–140, (1999).

[36] Wilson, E. O. *Zoogeography of Caribbean Insects: The biogeography of the West Indian ants (Hymenoptera: Formicidae).* Cornell University Press, Ithaca, NY, (1988).

[37] Wilson, E. O. Ants of the Dominican amber (Hymenoptera: Formicidae). 2. The first fossil army ants. *Psyche*, **92**, 11–16, (1985).

[38] Ward, P. S. Ants of the genus *Pseudomyrmex* (Hymenoptera: Formicidae) from Dominican amber, with a synopsis of the extant Antillean species. *Psyche*, **99**, 55–85, (1992).

[39] Baroni Urbani, C. and De Andrade, M. L. First description of fossil Dacetini ants with a critical analysis of the current classification of the tribe.(Amber Collection Stuttgart: Hymenoptera, Formicidae. VI: Dacetini.). *Stuttgarter Beitraege zur Naturkunde Serie B*, **198**, 1–65, (1994).

[40] Baroni Urbani, C. and De Andrade, M. L. The ant tribe Dacetini: limits and constituent genera, with descriptions of new species. *Annali del Museo Civico di Storia Naturale Giacomo Doria (Genova)*, **99**, 1–191, (2007).

[41] Dlussky, G. M. and Rasnitsyn, A. P. Ants (Insecta: Vespida: Formicidae) in the Upper Eocene amber of central and eastern Europe. *Paleontologicheskii Zhurnal*, **43**, 1024–1042, (2009).

[42] Baroni Urbani, C. First description of fossil gardening ants.(Amber collection Stuttgart and Natural History Museum Basel; Hymenoptera: Formicidae. I:

Attini.). *Stuttgarter Beitraege zur Naturkunde Serie B (Geologie und Palaeontologie)*, **54**, 1–13, (1980).

[43] Emery, C. Le formiche dell'ambra Siciliana nel Museo Mineralogico dell'Universit di Bologna. *Memorie della Accademia delle Scienze dell'Istituto di Bologna*, **5**, 141–165, (1891).

[44] Bolton, B. Taxonomy of the dolichoderine ant genus *Technomyrmex* Mayr (Hymenoptera: Formicidae) based on the worker caste. *Contributions of the American Entomological Institute*, **35**, 1–150, (2007).

[45] Carpenter, F. M. The fossil ants of North America. *Bulletin of the Museum of Comparative Zoology*, **70**, 1–66, (1930).

[46] Dlussky, G. M. The ant subfamilies Ponerinae, Cerapachyinae, and Pseudomyrmecinae (Hymenoptera, Formicidae) in the late Eocene ambers of Europe. *Paleontologicheskii Zhurnal*, **43**, 1043–1086, (2009).

[47] Dlussky, G. M. Genera of ants (Hymenoptera: Formicidae) from Baltic amber. *Paleontologicheskii Zhurnal*, **31**, 616–627, (1997).

[48] Wheeler, W. M. The Ants of the Baltic Amber. *Schriften der Physikalisch-Okonomischen Gesellschaft zu Konigsberg*, **55**, 1–142, (1915).

[49] Mayr, G. L. Die Ameisen des baltischen Bernsteins. *Beitrage zur Naturkunde Preussen*, **1**, 1–102, (1868).

[50] Dlussky, G. M. Ants of the genus *Dolichoderus* (Hymenoptera: Formicidae) from the Baltic and Rovno ambers. *Paleontologicheskii Zhurnal*, **36**, 50–63, (2002).

[51] Dlussky, G. The Ants of the Genus Lasius (Hymenoptera, Formicidae) from Late Eocene European Ambers. *Vestnik zoologii*, **45**, 14–27, (2011).

[52] Dlussky, G. M. and Radchenko, A. *Pristomyrmex rasnitsyni* sp. n., the first known fossil species of the ant genus *Pristomyrmex* Mayr (Hymenoptera, Formicidae) from the late Eocene Danish Amber. *Russian Entomological Journal*, **20**, 251–254, (2011).

[53] De Andrade, M. L. and Baroni Urbani, C. The Baltic amber ant genus *Bradoponera* (Hymenoptera: Formicidae), with description of two new species and a reassessment of the Proceratiini genera. *Revue Suisse de Zoologie*, **110**, 913–938, (2003).

[54] Branstetter, M. G. Origin and diversification of the cryptic ant genus *Stenamma* Westwood (Hymenoptera: Formicidae), inferred from multilocus molecular data, biogeography and natural history. *Systematic Entomology*, **37**, 478–496, (2012).

[55] Poinar, G., Archibald, B., and Brown, A. New amber deposit provides evidence of Early Paleogene extinctions, paleoclimates, and past distributions. *The Canadian Entomologist*, **131**, 171–177, (1999).

[56] Engel, M. S. and Grimaldi, D. A. Primitive new ants in cretaceous amber from Myanmar, New Jersey, and Canada (Hymenoptera: Formicidae). *American Museum Novitates*, **3485**, 1–24, (2005).

[57] Darling, D. C. and Sharkey, M. J. Insects from the Santana Formation, Lower Cretaceous, of Brazil: Order Hymenoptera. *Bulletin of the American Museum of Natural History*, **195**, 123–153, (1990).

[58] Grimaldi, D. and Engel, M. S. *Evolution of the Insects*. Cambridge University Press, (2005).

[59] Grimaldi, D. and Agosti, D. A formicine in New Jersey Cretaceous amber (Hymenoptera: Formicidae) and early evolution of the ants. *Proceedings of the National Academy of Sciences*, **97**, 13678–13683, (2000).

[60] Dlussky, G. M. New ants (Hymenoptera, Formicidae) from Canadian amber. *Paleontologicheskii Zhurnal*, **33**, 409–412, (1999).

[61] Antropov, A. V. Digger wasps (Hymenoptera, Sphecidae) in Burmese amber. *Bulletin of the Natural History Museum Geology Series*, **56**, 59–78, (2000).

[62] Zhang, H., Rasnitsyn, A. P., and Zhang, J. The oldest known scoliid wasps (Insecta, Hymenoptera, Scoliidae) from the Jehol biota of western Liaoning, China. *Cretaceous Research*, **23**, 77–86, (2002).

[63] Rasnitsyn, A. P., Jarzembowski, E. A., and Ross, A. J. Wasps (Insecta: Vespida= Hymenoptera) from the Purbeck and Wealden (Lower Cretaceous) of southern England and their biostratigraphical and palaeoenvironmental significance. *Cretaceous Research*, **19**, 329–391, (1998).

[64] Paradis, E., Claude, J., and Strimmer, K. APE: Analyses of Phylogenetics and Evolution in R language. *Bioinformatics*, **20**, 289–290, (2004).

[65] Rambaut, A., Suchard, M. A., Xie, D., and Drummond, A. J. Tracer v1.6. Available from http://beast.bio.ed.ac.uk/tracer, (2014).

[66] Guénard, B., Perrichot, V., and Economo, E. P. Integration of global fossil and modern biodiversity data reveals dynamism and stasis in ant macroecological patterns. *Journal of Biogeography*, **42**, 2302–2312, (2015).

[67] Gavryushkina, A., Welch, D., Stadler, T., and Drummond, A. J. Bayesian inference of sampled ancestor trees for epidemiology and fossil calibration. *PLoS Computational Biology*, **10**, e1003919, (2014).

[68] Revell, L. J. phytools: an R package for phylogenetic comparative biology (and other things). *Methods in Ecology and Evolution*, **3**, 217–223, (2012).

[69] Rabosky, D. L. and Goldberg, E. E. Model inadequacy and mistaken inferences of trait-dependent speciation. *Systematic Biology*, **64**, 340–355, (2015).

[70] Alfaro, M. E., Santini, F., Brock, C., Alamillo, H., Dornburg, A., Rabosky, D. L., Carnevale, G., and Harmon, L. J. Nine exceptional radiations plus high turnover explain species diversity in jawed vertebrates. *Proceedings of the National Academy of Sciences*, **106**, 13410–13414, (2009).

[71] May, M. R. and Moore, B. R. How well can we detect lineage-specific diversification-rate shifts? A simulation study of sequential AIC methods. *Systematic Biology*, **65**, 1076–1084, (2016).

[72] Rabosky, D. L. Automatic detection of key innovations, rate shifts, and diversity-dependence on phylogenetic trees. *PLoS ONE*, **9**, e89543, (2014).

[73] Moore, B. R., Hohna, S., May, M. R., Rannala, B., and Huelsenbeck, J. P. Critically evaluating the theory and performance of Bayesian analysis of macroevolutionary mixtures. *Proceedings of the National Academy of Sciences*, **113**, 9569–74, (2016).

[74] Rabosky, D. L., Mitchell, J. S., and Chang, J. Is BAMM flawed? Theoretical and practical concerns in the analysis of multi-rate diversification models. *Systematic Biology*, **66**, 477–498, (2017).

[75] Rabosky, D. L., Grundler, M., Anderson, C., Title, P., Shi, J. J., Brown, J. W., Huang, H., and Larson, J. G. BAMMtools: an R package for the analysis of evolutionary dynamics on phylogenetic trees. *Methods in Ecology and Evolution*, **5**, 701–707, (2014).

[76] Pennell, M. W., Eastman, J. M., Slater, G. J., Brown, J. W., Uyeda, J. C., FitzJohn, R. G., Alfaro, M. E., and Harmon, L. J. geiger v2.0: an expanded suite of methods for fitting macroevolutionary models to phylogenetic trees. *Bioinformatics*, **30**, 2216–2218, (2014).

[77] Grafen, A. The phylogenetic regression. *Philosophical Transactions of the Royal Society B: Biological Sciences*, **326**, 119–157, (1989).

[78] Rabosky, D. L. and Huang, H. A robust semi-parametric test for detecting trait-dependent diversification. *Systematic Biology*, **65**, 1–13, (2015).

[79] Ward, P. S. The phylogeny and evolution of ants. *Annual Review of Ecology, Evolution, and Systematics*, **45**, 23–43, (2014).

[80] Uyeda, J. C., Harmon, L. J., and Blank, C. E. A comprehensive study of cyanobacterial morphological and ecological evolutionary dynamics through deep geologic time. *PLoS ONE*, **11**, e0162539, (2016).
