# Supplementary material for: Macroecology and macroevolution of the latitudinal diversity gradient in ants
Source: Nat Commun. 2018 May 3;9:1778. doi: 10.1038/s41467-018-04218-4 (PMC5934361; doi:10.1038/s41467-018-04218-4)
Supplement: Supplementary file 2 — Description of Additional Supplementary Files [file 41467_2018_4218_MOESM2_ESM.pdf]

Description of Additional Supplementary Files

File Name: Supplementary Data 1

Description: Extant and fossil taxa included in the backbone phylogenetic analyses along with Genbank Accession codes for each molecular sequence.

File Name: Supplementary Data 2

Description: Monophyly constraints imposed during the Fossilized Birth-Death Process dating analysis in BEAST2.

File Name: Supplementary Data 3

Description: List of terminal clades and species counts used during the grafting procedures.
